# Recapitulating Cholangiopathy-Associated Necroptotic Cell Death In Vitro Using Human Cholangiocyte Organoids

**DOI:** 10.1016/j.jcmgh.2021.10.009

**Published:** 2021-10-23

**Authors:** Shaojun Shi, Monique M.A. Verstegen, Henk P. Roest, Arif I. Ardisasmita, Wanlu Cao, Floris J.M. Roos, Petra E. de Ruiter, Marije Niemeijer, Qiuwei Pan, Jan N.M. IJzermans, Luc J.W. van der Laan

**Affiliations:** 1 Department of Surgery, Erasmus MC Transplant Institute, Erasmus MC University Medical Center, Rotterdam, The Netherlands; 2 Department of Metabolic Diseases, Wilhelmina Children’s Hospital, University Medical Centre Utrecht, Utrecht, The Netherlands; 3 Department of Gastroenterology and Hepatology, Erasmus MC University Medical Center, Rotterdam, The Netherlands; 4 Department of Oncology, Shanghai East Hospital, Tongji University, Shanghai, P. R. China; 5 Division of Drug Discovery and Safety, Leiden Academic Centre for Drug Research, Leiden University, Leiden, The Netherlands

**Keywords:** Necroptosis, Apoptosis, Liver Disease, Biliary Injury, ALD, alcoholic liver disease, CTR, control, DAMP, damage-associated molecular pattern, DMEM, Dulbecco’s modified Eagle medium, ERCP, endoscopic retrograde cholangiopancreatography, hICO, intrahepatic cholangiocyte organoid derived from human donor liver, ICO, intrahepatic cholangiocyte organoid, LDLT, living donor liver transplantation, mICO, murine intrahepatic cholangiocyte organoid, MLKL, mixed lineage kinase domain-like, NASH, nonalcoholic steatohepatitis, Nec-1, necrostatin-1, Nec-1s, 7-Cl-O-necrostatin-1, NF-κB, nuclear factor-κB, NSA, necrosulfonamide, PBC, primary biliary cholangitis, PSC, primary sclerosing cholangitis, PI, propidium iodide, p-IKKα/β, phosphorylated inhibitory-κB kinase α/β, pMLKL, phosphorylated mixed lineage kinase domain-like, POA, palmitoleic acid, RIPK, receptor-interacting protein, S, Smac mimetic, Smac, second mitochondria-derived activator of caspases, T, tumor necrosis factor-, TEM, transmission electron microscopy, TNF-α, tumor necrosis factor-α, Z, Z-VAD-FMK

## Abstract

**Background & Aims:**

Liver and bile duct diseases often are associated with extensive cell death of cholangiocytes. Necroptosis represents a common mode of programmed cell death in cholangiopathy, however, detailed mechanistic knowledge is limited owing to the lack of appropriate in vitro models. To address this void, we investigated whether human intrahepatic cholangiocyte organoids (ICOs) can recapitulate cholangiopathy-associated necroptosis and whether this model can be used for drug screening.

**Methods:**

We evaluated the clinical relevance of necroptosis in end-stage liver diseases and liver transplantation by immunohistochemistry. Cholangiopathy-associated programmed cell death was evoked in ICOs derived from healthy donors or patients with primary sclerosing cholangitis or alcoholic liver diseases by the various stimuli.

**Results:**

The expression of key necroptosis mediators, receptor-interacting protein 3 and phosphorylated mixed lineage kinase domain-like, in cholangiocytes during end-stage liver diseases was confirmed. The phosphorylated mixed lineage kinase domain-like expression was etiology-dependent. Gene expression analysis confirmed that primary cholangiocytes are more prone to necroptosis compared with primary hepatocytes. Both apoptosis and necroptosis could be specifically evoked using tumor necrosis factor α and second mitochondrial-derived activator of caspases mimetic, with or without caspase inhibition in healthy and patient-derived ICOs. Necroptosis also was induced by ethanol metabolites or human bile in ICOs from donors and patients. The organoid cultures further uncovered interdonor variable and species-specific drug responses. Dabrafenib was identified as a potent necroptosis inhibitor and showed a protective effect against ethanol metabolite toxicity.

**Conclusions:**

Human ICOs recapitulate cholangiopathy-associated necroptosis and represent a useful in vitro platform for the study of biliary cytotoxicity and preclinical drug evaluation.

## Graphical abstract

**Figure:**
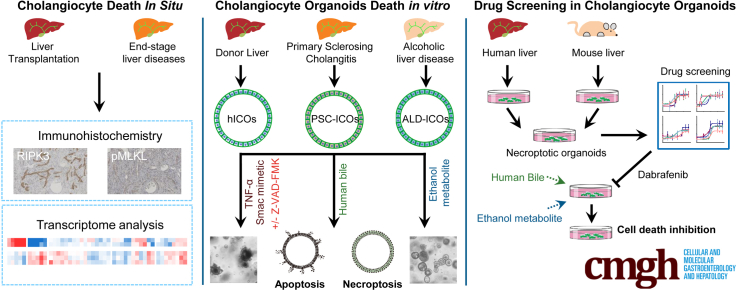

**Summary**

We successfully recapitulated cholangiopathy-associated necroptosis using human cholangiocyte organoids. To some extent, this new model can contribute to a better understanding of cholangiopathy pathogenesis and provide insights for the future development of therapeutics.

Cholangiocytes, lining the biliary epithelium, are damaged primarily during liver injury (cholangiopathies), which often causes advanced liver failure and represents an unmet need in clinical medicine. Cholangiopathies are featured by biliary obstruction, ductopenia, biliary hyperplasia, inflammation, and fibrosis, and can be evoked by various endogenous or exogenous signals/stimuli.^1^ The actual death of biliary epithelium, by either apoptosis or necrosis, serves as one of the core pathogenic mechanisms in cholangiopathies, but is not well characterized. Apoptosis of cholangiocytes has been investigated intensively to be a common death mode in cholangiopathies, and could be caused by immunologic factors, exogenous toxins, or endogenous bile salts.^2^ It is well known that the cell death process is associated strongly with inflammation in human diseases,^3^ while apoptosis generally is regarded as a noninflammatory cell death type.^4^ This raises the possibility that other cell death types, characterized with immunogenetic potential, are involved in cholangiopathies pathogenesis.

Necroptosis is an emerging type of programmed cell death that incorporates the features of apoptosis and necrosis and has been reported to be involved in various liver diseases.^5^ Necroptosis induced by activation of the specific cell death receptors shares the same upstream molecular machinery with extrinsic apoptosis. The receptor-interacting protein kinase 1 (RIPK1) and RIPK3 represent the critical kinases determining whether apoptosis or necroptosis is induced. In short, RIPK1 and RIPK3 are cleaved by caspase 8 in the setting of apoptosis, while inhibition of caspase 8 or its adaptor, Fas-associated via death domain, leads to the formation of RIPK1/RIPK3 complex and necroptotic signal activation.^5^ Subsequent phosphorylation of mixed lineage kinase domain-like (MLKL) initiates MLKL oligomerization, membrane translocation, and membrane rupture, which is widely recognized as the hallmark of necroptosis.^6^ As a result of cellular leakage, the passive release of the damage-associated molecular patterns (DAMPs), associated particularly with necroptosis, contributes to inflammation responses, known as sterile inflammation or necroinflammation. In addition to DAMPs, necroptosis also shows strong immunogenic capacity by mediating the active paracrine and autocrine of cytokines and chemokines, promoting inflammation in a cell-intrinsic manner.^7^ The cytokine tumor necrosis factor-α (TNF-α) is a potent inducer of inflammation-associated cell death in biliary pathogenesis.^8^

Increasing evidence has shown strong links between necroptosis and cholangiopathy pathogenesis. The unique role of necroptosis in cholangiocytes has been unraveled recently but remains a matter of intense debate. Activation of RIPK3-dependent necroptosis is the core event in primary biliary cholangitis (PBC) and experimental cholestasis.^9^ In a murine model of spontaneous liver injury, RIPK3-mediated necroptosis mainly drives biliary damage, while apoptosis mainly drives hepatocellular damage. This also has been confirmed further in vitro that mouse cholangiocytes can undergo necroptosis while hepatocytes appear to be resistant to necroptosis stimulation but can exclusively undergo apoptosis.^10^ An exclusive overexpression of RIPK3 in cholangiocytes has been proven histologically in murine and human nonalcoholic steatohepatitis (NASH) livers.^11^ This difference between hepatocytes and cholangiocytes is possibly owing to the availability of RIPK3.^9^^,^^10^ Furthermore, the necroptosis-associated cytokine microenvironment could determine the lineage commitment of liver tumorigenesis by switching hepatocellular carcinoma to cholangiocarcinoma development.^12^

The underlying necroptotic mechanisms have been broadly studied using human and mouse cell lines. However, the species and inter–cell line differences make these models suboptimal.^13^ To improve our understanding of the role of necroptosis in cholangiopathies, better in vitro models recapitulating key physiological aspects of the human cholangiocellular system are needed. The organoid culture represents a near-physiological system for human disease modeling in vitro. Recent studies have applied intestinal organoids to mimic necroptosis pathways, linked to inflammatory bowel disease's etiology, in vitro.^14^ We and others previously described methods to culture and expand human and mouse cholangiocyte organoids, which are capable of self-organization and retain most biliary characteristics in vitro.^15^, ^16^, ^17^

The present study aims to investigate necroptosis pathways in intrahepatic cholangiocyte organoids (ICOs) as a model for cholangiopathy-associated programmed cell death. We showed that RIPK3 is expressed predominantly in cholangiocytes, rather than hepatocytes, in liver biopsy specimens from patients with end-stage liver diseases and donor livers in liver transplantation. TNF-α–induced necroptosis signaling could be recapitulated using ICOs derived from human donor liver (hICOs), ICOs derived from patients with alcoholic liver disease (ALD-ICOs), and ICOs derived from patients with primary sclerosing cholangitis (PSC-ICOs). Ethanol- and bile-associated toxic insults specifically induced necroptosis in both donor- and patient-derived ICOs, in a time- and dose-dependent manner. The hICOs could be used for drug screening, uncovering interdonor variable and species-specific responses to cell death inhibitors, and identifying dabrafenib as a potent necroptosis inhibitor.

## Results

### Up-Regulation of Necroptosis Effector Proteins During Liver and Bile Duct Injury

**Figure 1:**
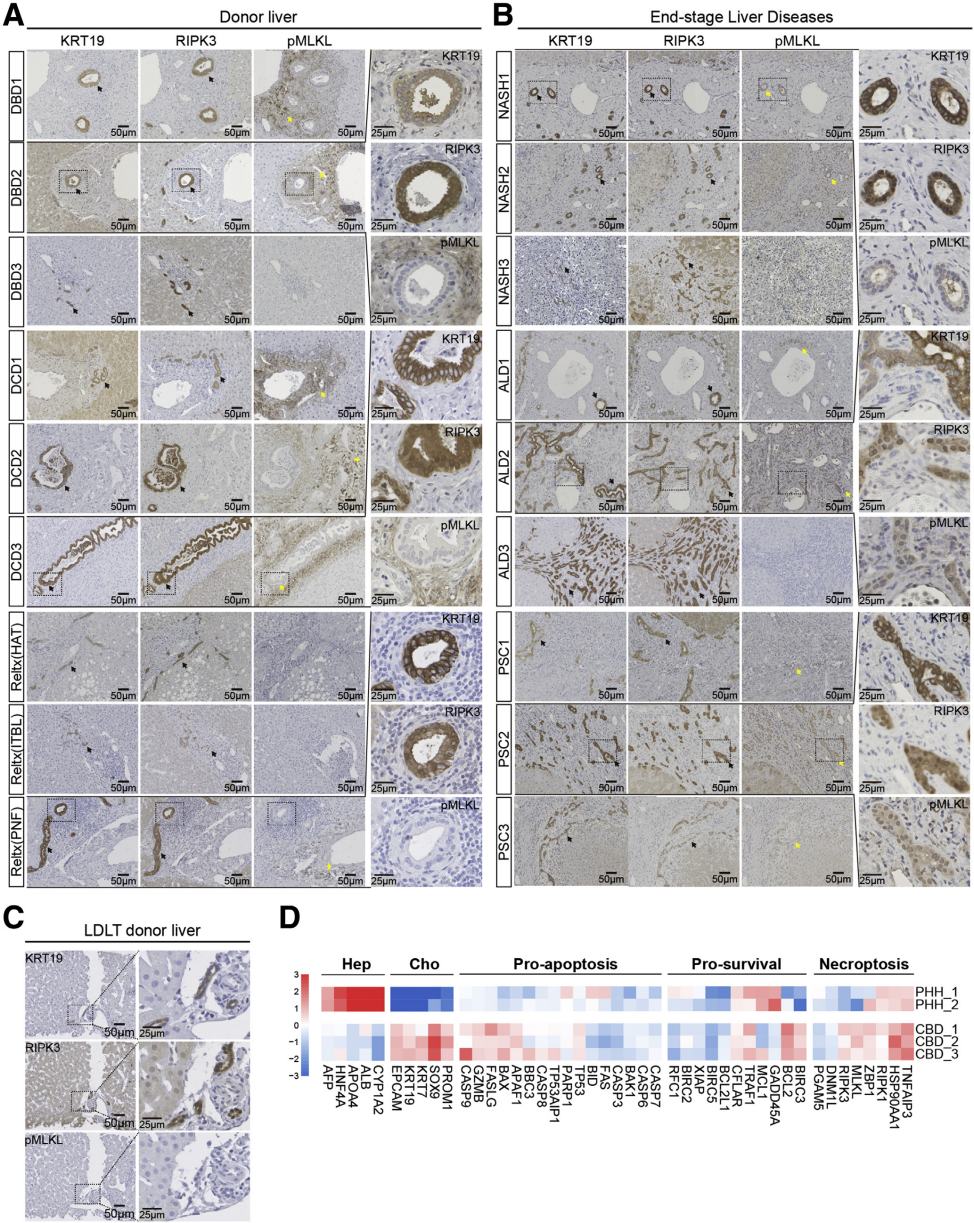
**Necroptotic mediators are associated with hepatic pathogenesis.** (*A*) Representative microscopic images of immunohistochemical staining with cytokeratin-19 (cholangiocyte marker) and RIPK3 (*black arrows*), as well as pMLKL (*yellow arrows*), on consecutive sections of biopsy specimens from liver transplantation, including donation after brain death (DBD) (n = 3) and donation after cardiac death (DCD) (n = 3) donor livers and explant livers from recipients undergoing liver retransplantation as a result of hepatic artery thrombosis (HAT), ischemic-type biliary lesions (ITBL) (n = 1), and primary graft nonfunction (PNF) (n = 1). Detailed images are shown in the *right panel* (magnification, 800×). (*B*) Sections from patients with end-stage liver diseases, including NASH (n = 3), ALD (n = 3), and PSC (n = 3) are shown. (*C*) Sections from nonischemic liver tissue obtained from a living donor (LDLT) are shown (n = 1). All detailed images are shown in the *right panel* (magnification, 800×). (*D*) Expression heatmap of selected genes associated with hepatocyte (Hep), cholangiocyte (Cho), pro-apoptosis, prosurvival, and necroptosis machinery in primary cholangiocytes derived from the common bile duct (CBD; n = 4) and primary human hepatocytes (PHHs; n = 2). KRT19, cytokeratin-19.

To analyze the clinical relevance of necroptotic mediators in liver transplantation and end-stage liver diseases, immunohistochemistry was performed on consecutive liver slices using validated RIPK3^18^^,^^19^ and phosphorylated mixed lineage kinase domain-like (pMLKL)^20^^,^^21^ antibodies. A cholangiocyte-specific cytokeratin-19 antibody was applied to localize bile ducts. Basal expression of nuclear RIPK3 protein in cholangiocytes was found in a healthy and ischemia-free liver biopsy specimen from a living donor liver transplantation (LDLT) (Figure 1*C*). Predominant cytoplasmic RIPK3 expression was found in cholangiocytes in donor livers with ischemia-reperfusion injury and in patients with end-stage liver diseases. Only relatively limited expressed of RIPK3 was observed in hepatocytes in some biopsy specimens (Figure 1*A* and *B*). RIPK3 expression was observed in cholangiocytes from small and bigger bile ducts and from fibrotic areas showing ductal reactivity (Figure 1*B*). Immunostaining for necroptosis executor protein, pMLKL, in both donation after cardiac death and donation after brain death donor livers showed only positivity in nonparenchymal cells in the portal triad, but not in cholangiocytes or hepatocytes (Figure 1*A*). pMLKL positivity was absent in the LDLT donor liver (Figure 1*C*). However, in some patients with end-stage liver diseases, clear but moderate positivity for pMLKL was observed in cholangiocytes (Figure 1*B*). These findings are in agreement with previous studies in experimental models^9^^,^^10^ and suggest that necroptotic mediators are involved in cholangiopathy but vary between different liver disease etiologies. Transcriptome analysis in a set of cell death- and survival-related genes in freshly isolated human primary hepatocytes^22^ and primary cholangiocytes (isolated from the common bile duct)^23^ are shown in Figure 1*D*. We observed a difference in expression of apoptosis, survival, and necroptosis genes between hepatocytes and cholangiocytes. These different profiles confirm that cholangiocytes have a higher expression of genes related to necroptosis cell death. Of note, primary cholangiocytes up-regulated RIPK3 and MLKL messenger RNA compared with hepatocytes (Figure 1*D*). Taken together, these findings suggest that cholangiocytes up-regulate the necroptosis machinery and are prone to necroptotic cell death in various pathologic conditions.

### Extrinsic Activation by TNF-α Induces Necroptotic Cell Death in Donor- and Patient-Derived ICOs

**Figure 2:**
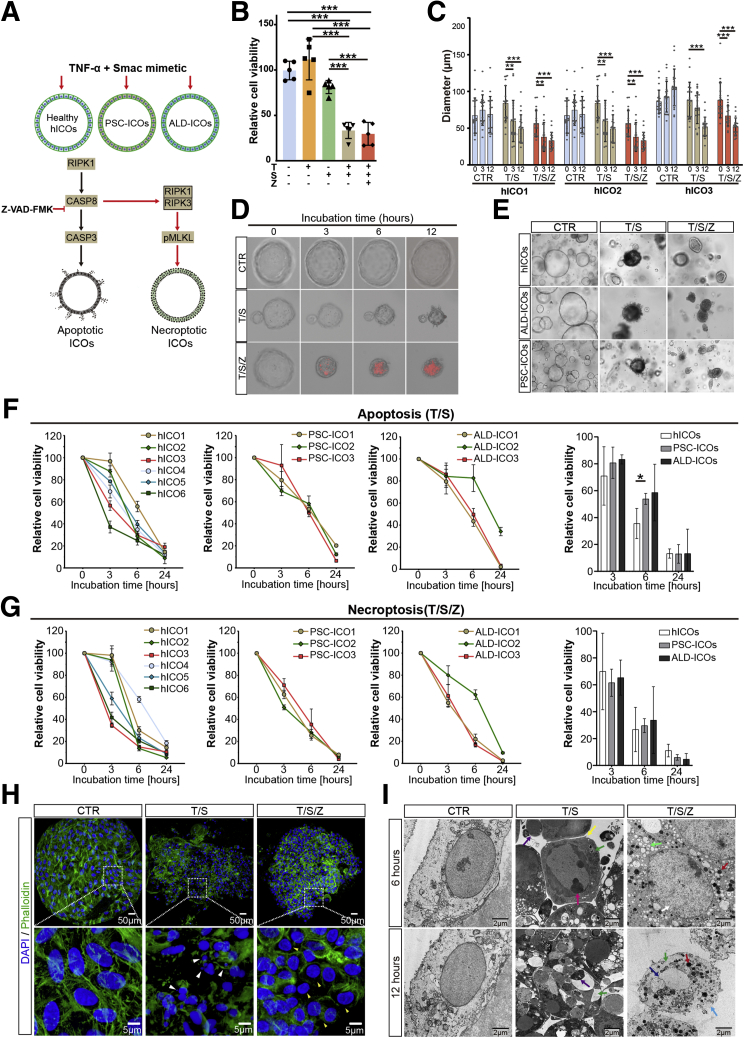
**Distinct features of necroptotic cell death in hICOs.** (*A*) Apoptosis and necroptosis were induced in hICOs, PSC-ICOs, and ALD-ICOs using different combinations of TNF-α (T, 20 ng/mL) and Smac mimetic (S, 120 μmol/L), with or without Z-VAD-FMK (Z, 50 μmol/L). (*B*) Cell viability measurement was performed using CellTiter-Glo reagent. Relative cell viability was calculated by normalizing to dimethyl sulfoxide–treated hICOs (CTR) after 6 hours of incubation (n = 5). (*C*) The diameter of stimulated hICOs was determined in a time-course manner and analyzed using ImageJ software. (*D*) Real-time, live-cell imaging of stimulated hICOs incubated with 100 ug/mL PI (red). (*E*) Representative bright-field images of stimulated hICOs, ALD-ICOs, and PSC-ICOs after 6 hours. (*F* and *G*) Time-course cell viability of stimulated hICOs (n = 6), ALD-ICOs (n = 3), and PSC-ICOs (n = 3). (*H*) Confocal imaging of the hICOs treated for 6 hours after staining with 4′,6-diamidino-2-phenylindole (DAPI)/phalloidin. Enlarged images from the *boxed area* are shown in the *bottom panel*. (*I*) Representative TEM images of stimulated hICOs at a time point of 6 and 12 hours. Typical cell death–related characteristics were found as indicated by *arrows* (pink, pyknosis; dark green, shrunken cytoplasm; purple, fragmented nucleus; yellow, plasma membrane blebbing; light green, cytoplasmic vacuolization; red, condensed mitochondria; dark blue, karyolysis; light blue, ruptured plasm membrane; white, rounded nuclei). Data are means ± SD. ∗*P* < .05, ∗∗*P* < .01, ∗∗∗*P* < .001 by (*C*, *F*, *G*) Mann–Whitney test and (*B*) the Kruskal–Wallis test followed by the Dunn post hoc test.

Inflammation and cell death are at the heart of cholangiopathies and largely dependent on TNF-α signaling.^8^ To induce extrinsic necroptosis, hICOs were treated with an established combination of TNF-α (T), second mitochondria-derived activator of caspases (Smac) mimetic (S), an antagonist of inhibitor of apoptosis proteins, and the pan-caspase inhibitor Z-VAD-FMK (Z), although T/S treatment theoretically could promote extrinsic apoptosis^6^ (Figure 2*A*). Combined treatment with TNF-α and Smac mimetic (T/S) triggered more than 50% loss of cell viability in hICOs (n = 5; *P* < .001), whereas cell viability could not be rescued by the supplement of pan-caspase inhibitor Z-VAD-FMK (T/S/Z) (n = 5; *P* < .001) (Figure 2*B*). Both T/S- and T/S/Z-exposed hICOs showed a time-dependent and significantly decreased organoid size (Figure 2*C*). Of note, at the terminal stage of cell death, T/S-exposed hICOs turned dark and completely disrupted, while T/S/Z-exposed hICOs retained a shrunken but gray and intact lumen (Figure 2*D* and *E*). As shown in Figure 2*D* and *E*, cells shed from the lumen of the organoids upon T/S treatment and showed a weak propidium iodide (PI) intake. On the contrary, in the T/S/Z condition, few cells shed from the lumen, but strong PI-positivity cells were observed, implying loss of plasma membrane integrity.

To determine whether liver disease etiologies affect the response of ICOs to cell death induction, ALD-ICOs and PSC-ICOs were cultured and stimulated as indicated previously. Both donor- and patient-derived ICOs shared similar morphologic features after apoptosis and necroptosis induction. Noticeably, some interdonor variations in hICOs were observed at 3 and 6 hours after cell death induction, but at 24 hours all hICOs showed more than 80% cell death (Figure 2*F* and *G*). Obvious interpatient variations were observed in ALD-ICOs, but not in PSC-ICOs, in which ALD-ICO2 showed a relative resistance to both apoptosis and necroptosis induction (Figure 2*E* and *F*). Moreover, apoptosis appeared to be delayed in ALD-ICOs at 6 hours, compared with hICOs (58.6% ± 21.05% vs 35.56% ± 11.16%; *P* < .05). Despite this, these donor- and patient-derived hICOs behaved similarly upon apoptosis and necroptosis stimuli.

To further confirm that the simulation with T/S/Z induces necroptotic cell death in hICOs, fluorescent and electron (transmission electron microscopy [TEM]) microscopy analysis was performed. As shown in Figure 2*H*, 4′,6-diamidino-2-phenylindole/phalloidin staining showed clear disintegration of the actin cytoskeleton in dying organoids. T/S/Z-exposed hICOs were featured by a small rounded and pyknotic nucleus as previously reported.^14^ In contrast, a massive fragmentation of the nucleus was observed in T/S-exposed hICOs, indicating an apoptosis-like phenotype. Phalloidin staining showed only limited degradation of the actin cytoskeleton in the T/S/Z condition, whereas clear actin loss was observed in the T/S condition. Moreover, TEM analysis showed a phenotypic appearance of pyknosis, shrunken cytoplasm, plasm membrane blebbing, and nucleus fragmentation in T/S-stimulated hICOs, representing apoptotic features^24^ (Figure 2*F*). Nevertheless, the T/S/Z-exposed hICOs showed rounded nucleus, karyolysis, cytoplasmic vacuolization, mitochondrial condensation, and ultimate cell membrane rupture, resembling a necroptosis-like phenotype (Figure 2*F*).^14^ It seems plausible that T/S/Z treatment could induce specific cell death resembling necroptosis.

**Figure 3:**
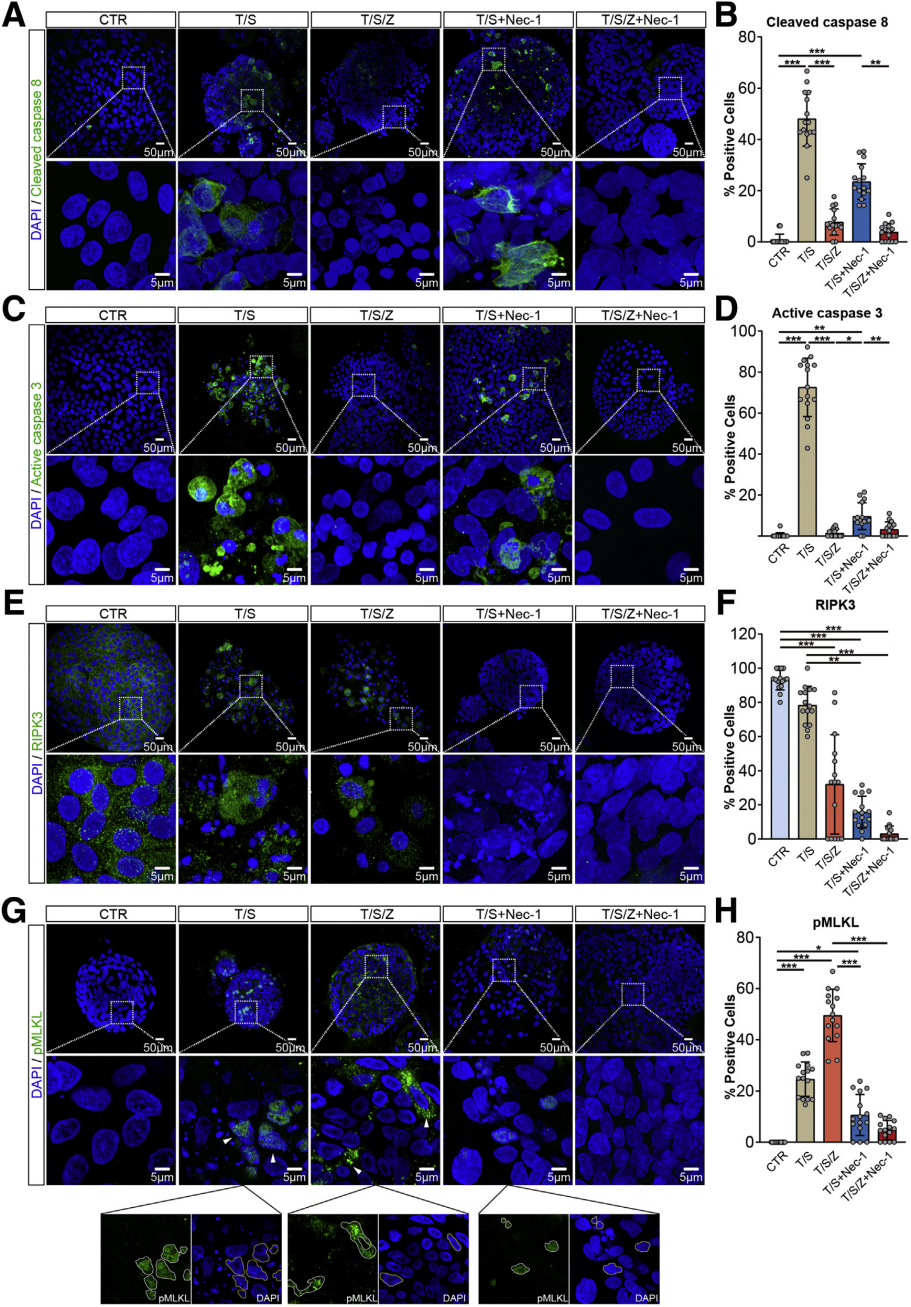
**Activation of caspases and key necroptosis mediators in TNF-α–stimulated hICOs.** (*A–H*) hICOs were treated as indicated for 6 hours, with or without co-incubation with Nec-1 (20 μmol/L). Stimulated hICOs were stained for (*A*) cleaved caspase 8, (*C*) active caspase 3, (*E*) RIPK3, and (*G*) pMLKL. (*A*, *C*, *E*, and *G*) Representative fluorescent images are shown. Enlarged images from the *boxed area* are shown below. (*G*) Images with individual colors are shown below the indicated merged images (magnification, 400×). 4′,6-diamidino-2-phenylindole (DAPI) and pMLKL staining are marked by yellow and white outlines separately. (*B*, *D*, *F*, and *H*) Quantification of positive cells in fluorescent images was performed using ImageJ (at least 15 high-power fields in each condition). Data are means ± SD. (*B*, *D*, *F*, and *H*) ∗*P* < .05, ∗∗*P* < .01, ∗∗∗*P* < .001, Kruskal–Wallis test followed by the Dunn post hoc test.

**Figure 4:**
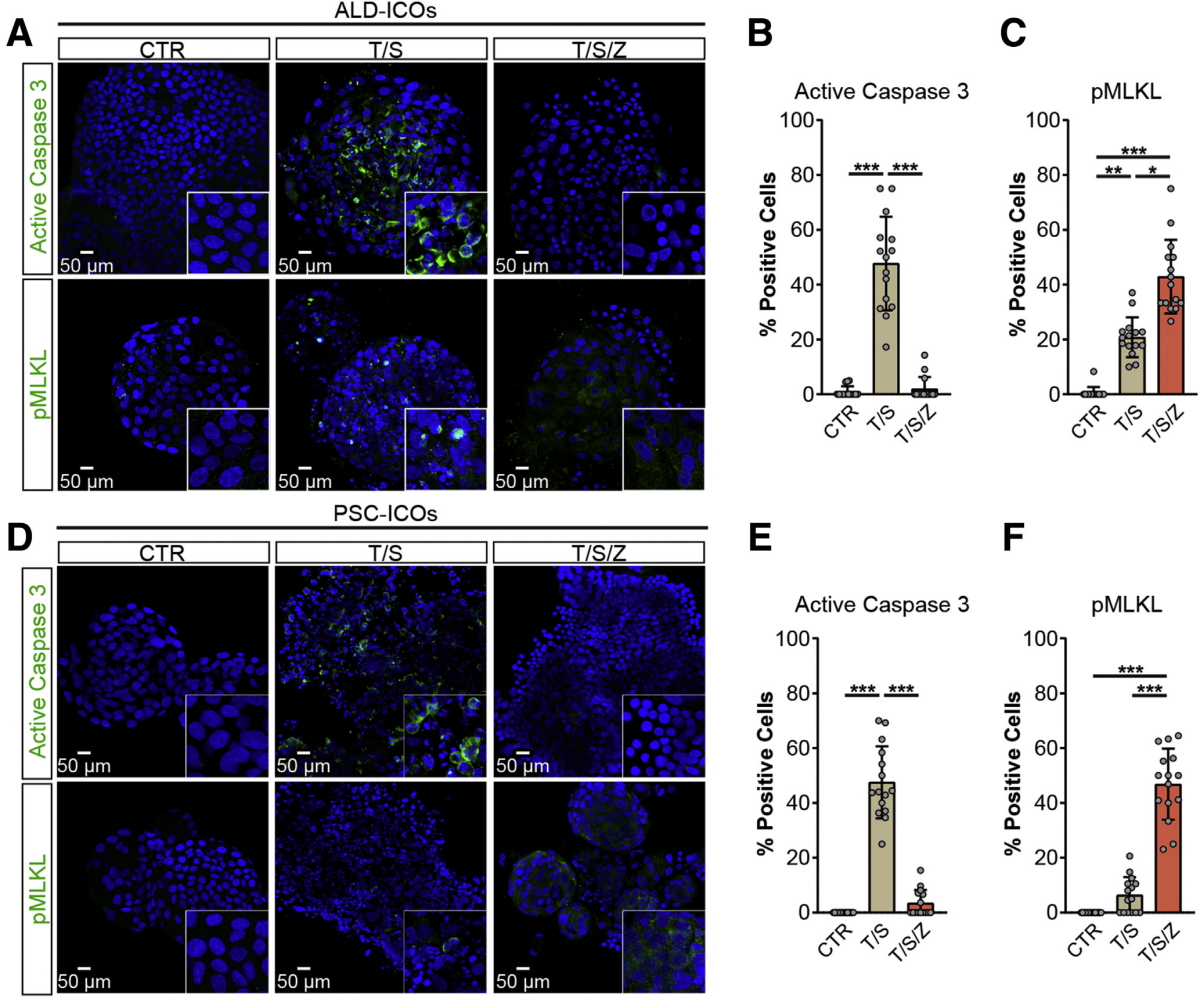
**Activation of key apoptosis and necroptosis mediators in TNF-α–stimulated ALD-ICOs and PSC-ICOs.** (*A–F*) ALD-ICOs (n = 3) and PSC-ICOs (n = 3) were treated as indicated for 6 hours and then stained for active caspase 3 and pMLKL. (*A* and *D*) Representative fluorescent images are shown. (*B*, *C*, *E*, and *F*) Quantification of positive cells in fluorescent images was performed using ImageJ (at least 15 high-power fields in each condition). Data are means ± SD. (*B*, *C*, *E*, and *F*) ∗*P* < .05, ∗∗*P* < .01, ∗∗∗*P* < .001, Kruskal–Wallis test followed by the Dunn post hoc test.

To further define the induced cell death, immunostaining for key apoptosis and necroptosis mediators was conducted. Necrostatin-1 (Nec-1), a well-known necroptosis inhibitor, also was administered in stimulated hICOs to inhibit induced cell death. As shown in Figure 3*A–D*, clear activation of caspase 8 and caspase 3 was observed in hICOs stimulated with T/S (*P* < .001), suggesting the induction of extrinsic apoptosis in these cells. Supplementing with caspase inhibitor Z-VAD-FMK prevented the activation of both caspase 3 and 8 (*P* < .001). The addition of Nec-1 to the T/S condition did not show a significant inhibitory effect on caspase 8 activity (*P* = .62) (Figure 3*A* and *B*), and only slightly suppressed caspase 3 activity (*P* = .08) (Figure 3*C* and *D*). Moreover, RIPK3 was highly expressed in control (CTR) and was down-regulated by T/S/Z (*P* < .001) treatment, but could not be restored by Nec-1, possibly resulting from RIPK3 phosphorylation upon stimulation^25^ (Figure 3*E* and *F*). The induction of necroptosis was confirmed further by the apparent cytosolic and membrane translocation of pMLKL, a typical necroptosis phenotype in T/S/Z-treated hICOs and could be entirely reduced by Nec-1 (*P* < .001) (Figure 3*G* and *H*). Intriguingly, we also found that pMLKL accumulated exclusively in the nucleus of T/S-exposed hICOs and could be minimized slightly by Nec-1 (*P* = .21), which is in line with previous studies^26^ (Figure 3*G*). Similar expression patterns of active caspase 3 and pMLKL also were found in ALD-ICOs (Figure 4*A–C*) and PSC-ICOs (Figure 4*D–F*) after T/S or T/S/Z treatment. To summarize, these findings confirm that necroptosis could be induced in human ICOs by TNF-α and Smac mimetic when caspase activity is suppressed.

### Necroptotic hICOs Show Different Nuclear Factor-κB Signaling and Transcriptional Activation of Inflammatory Genes

**Figure 5:**
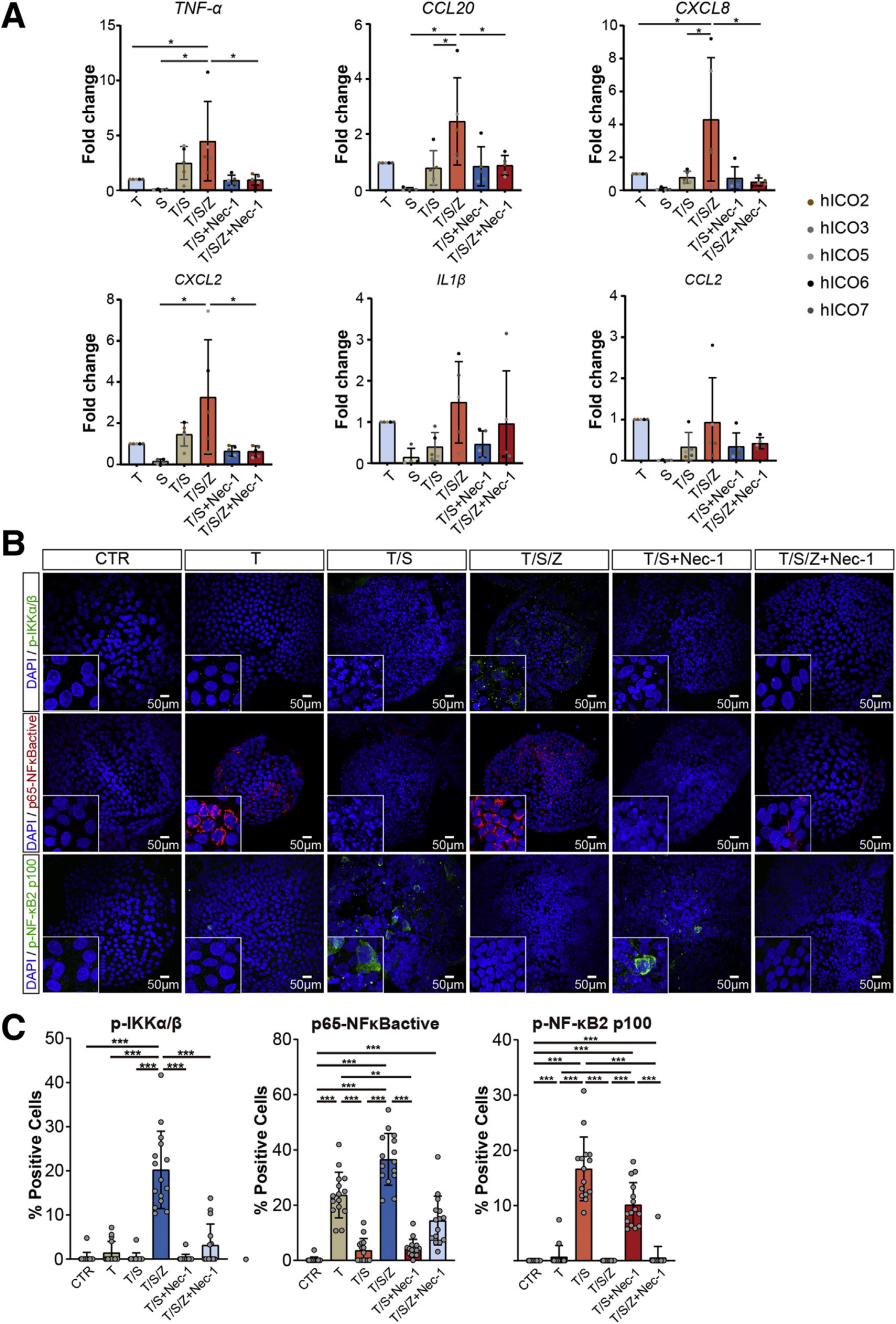
**Transcriptional profiles of inflammatory genes and NF-κB signaling in necroptotic hICOs.** hICOs were stimulated as indicated for 6 hours. (*A*) Gene expression levels of inflammatory genes were measured by quantitative polymerase chain reaction (n = 5). Fold change relative to TNF-α–treated hICOs was presented. (*B*) Representative confocal images of p-IKKα/β, p–NF-κB2 p100, and p65–NF-κBactive staining in treated hICOs are shown. (*C*) Quantification of positive cells in fluorescent images was performed using ImageJ (at least 15 high-power fields in each condition). Data are means ± SD. ∗*P* < .05, ∗∗*P* < .01, ∗∗∗*P* < .001, (*A*) 1-way analysis of variance test followed by the Tukey post hoc test and (*C*) the Kruskal–Wallis test followed by the Dunn post hoc test. DAPI, 4′,6-diamidino-2-phenylindole.

It is well established that the proinflammatory nature of necroptosis is derived from the passive release of DAMPs and the active synthesis of necroptosis-associated cytokines and chemokines.^7^ The expression of necroptosis-associated inflammatory genes was examined in hICOs (n = 5). Given that TNF-α also represents a potent stimulator of cytokine production, the expression of inflammatory genes was normalized to hICOs treated with TNF-α alone. As shown in Figure 5*A*, necroptosis-associated genes, *CCL20* (*P* < .05) and *CXCL8* (*P* < .05), were up-regulated significantly in necroptotic ICOs compared with T/S conditions. Gene expression of *CXCL2* (*P* = .20), *CCL2* (*P* = .41), and *IL1β* (*P* = .19) showed up-regulation in necroptotic hICOs, but this did not reach statistical significance. Gene expression of the *TNF-α* gene itself was clearly up-regulated in necroptotic hICOs (*P* < .05), but not in the T/S condition (*P* = .76), compared with the T condition. Supplementing with Nec-1 in necroptotic, but not apoptotic, hICOs potently resulted in a clear and significant reduction in expression of *TNF-α*, *CCL20*, *CXCL8*, and *CXCL2* (Figure 5*A*). These data indicate that only necroptotic, but not apoptotic, hICOs up-regulate proinflammatory genes, which is consistent with the inflammatory nature of necroptosis.

The active synthesis of cytokines during necroptosis has been reported to be mediated by signaling of the canonical nuclear factor kappa B (NF-κB) pathway.^7^ In contrast, apoptosis involves activation of the noncanonical NF-κB pathway induced by TNF-α and Smac mimetic signaling.^27^ We reasoned that distinct activation of NF-κB signaling might be observed in apoptotic and necroptotic hICOs. To this end, phosphorylated inhibitory-κB kinase α/β (p-IKKα/β), p65–NF-κB_active_, and p–NF-κB2 p100 was determined by immunofluorescence. As shown in Figure 5*B* and *C*, we found that p-IKKα/β was expressed exclusively in necroptotic hICOs (*P* < .001) and could be prevented by Nec-1 (*P* < .001). In addition, activation of the canonical NF-κB signaling, as indicated by p65–NF-κB_active_ positivity, was seen in necroptotic (*P* < .001), but not in apoptotic, hICOs (Figure 5*B* and *C*), which could be slightly suppressed by Nec-1 (*P* = .09). hICOs stimulated with TNF-α alone also showed increased p65–NF-κB_active_ positivity (*P* < .001). Noncanonical NF-κB signaling indicated by p–NF-κB2 p100 was up-regulated in apoptotic (*P* < .001), but not necroptotic, hICOs, which could not be suppressed by Nec-1 (Figure 5*B* and *C*). Collectively, these results show that necroptotic-associated inflammatory genes are up-regulated in necroptotic hICOs and that NF-κB signaling is activated in necroptotic hICOs in divergent manners from apoptotic hICOs.

### Ethanol-Metabolite Induces Dose-Dependent Necroptosis in Human ICOs

**Figure 6:**
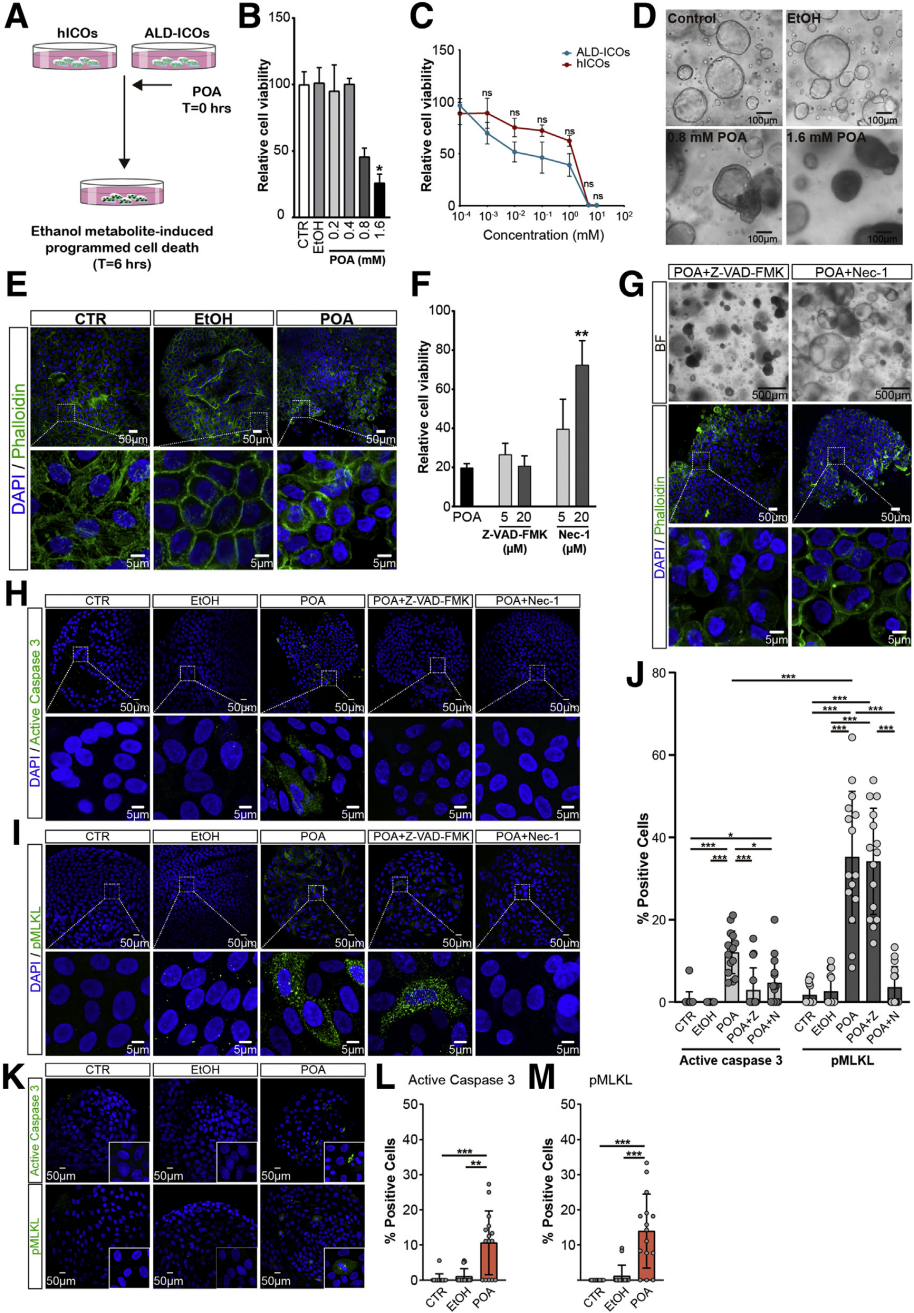
**Ethanol-metabolite induces dose-dependent necroptosis in ICOs.** (*A*) hICOs and ALD-ICOs were stimulated with POA for 6 hours. (*B*) hICOs were exposed to 2% ethanol (EtOH) or POA at different concentrations in EtOH. Cell viability was measured in the treated hICOs using CellTiter-Glo reagent. Relative cell viability was normalized to untreated hICOs (CTR) (n = 3). (*C*) hICOs and ALD-ICOs were stimulated with POA at different concentrations for 6 hours and cell viability was determined and analyzed as indicated. (*D*) Representative bright-field images of stimulated hICOs at 6 hours. (*E*) Confocal imaging of the treated hICOs after staining with 4′,6-diamidino-2-phenylindole (DAPI)/phalloidin. Enlarged images from the *boxed area* are shown in the *bottom panels*. (*F*) hICOs were pretreated with Z-VAD-FMK or Nec-1, at the indicated concentration 1 hour before POA treatment and then co-incubated with POA (1.6 mmol/L) for 6 hours. Cell viability was determined and presented as mentioned before. (*G–J*) hICOs were pretreated with Z-VAD-FMK (20 μmol/L) or Nec-1 (20 μmol/L), respectively, and then incubated with POA (1.6 mmol/L) as indicated previously. (*G*) Representative bright-field and confocal images (DAPI/phalloidin) of the treated hICOs are shown. Enlarged images from the *boxed area* are shown in the *bottom panel*. (*H* and *I*) Staining of active caspase 3 and pMLKL in stimulated hICOs are shown. (*J*) Quantification of positive cells in fluorescent images was performed using ImageJ (at least 15 high-power fields in each condition). Enlarged images from the *boxed area* are shown in the *bottom panels*. (*K–M*) ALD-ICOs (n = 3) were stimulated with 2% EtOH or POA (1.6 mmol/L) for 6 hours. (*K*) Staining of active caspase 3 and pMLKL in stimulated ALD-ICOs is shown. (*L* and *M*) Quantification of positive cells in fluorescent images was performed as indicated earlier. Data are means ± SD. ∗*P* < .05, ∗∗*P* < .01, ∗∗∗*P* < .001, (*B* and *F*) Mann–Whitney test, (*C*) 2-way analysis of variance post-Sidak multiple comparisons test, and (*J*, *L*, and *M*) Kruskal–Wallis test followed by the Dunn post hoc test. BF, bright field.

The toxicity of alcohol abuse on the liver is well known. These hepatotoxic effects are related mainly to exposure to breakdown products of ethanol metabolized in hepatocytes. Recent studies have indicate that not only hepatocytes but also cholangiocytes are affected by ethanol metabolites. Cholangiocytes play a previously unrecognized role in the development of cholestasis in alcoholic hepatitis.^28^ We tested ethanol-associated biliary injury in vitro, using hICOs and ALD-ICOs, which potentially are associated with alcoholic liver disease etiology (Figure 6*A*). The nonoxidative ethanol metabolite palmitoleic acid (POA) has been implicated as a critical mediator in ethanol-evoked epithelial cell injury.^29^ We found that POA dissolved in ethanol resulted in significantly reduced cell viability in hICOs in a concentration-dependent manner (n = 3; *P* < .05) (Figure 6*B* and *C*). Interestingly, we found that ALD-ICOs appeared to be more sensitive to POA toxicity, although the difference was not statistically significant (Figure 6*C*). POA-exposed hICOs showed an appearance resembling necroptosis as indicated by a shrunken gray, but intact, lumen; diminished cytoplasm; and rounded, but not fragmental, nucleus (Figure 6*D* and *E*). Treatment with Nec-1, rather than Z-VAD-FMK, could significantly rescue the POA-induced cell death, implying involvement of necroptosis (n = 4; *P* < .01) (Figure 6*F* and *G*). This was confirmed by significant up-regulation of pMLKL in POA-treated hICOs (n = 4; *P* < .01). The expression of pMLKL can be suppressed completely by Nec-1 (n = 4; *P* < .001) (Figure 6*I* and *J*). Of note, sporadic active caspase 3–positive cells also were observed in POA-exposed hICOs, but were significantly less than pMLKL positivity (12.09% ± 5.17% vs 35.20% ± 15.99%; *P* < .001), which could be inhibited by both Z-VAD-FMK (*P* < .001) and Nec-1 (*P* < .05) (Figure 6*G*, *H* and *J*). A significant increase of active caspase 3 and pMLKL also was observed in POA-treated ALD-ICOs (Figure 6*K*, *L* and *M*). Altogether, our results show that ethanol-metabolite–mediated necroptosis could be triggered in human ICOs.

### Human Bile-Induced Cell Death in Human ICOs

**Figure 7:**
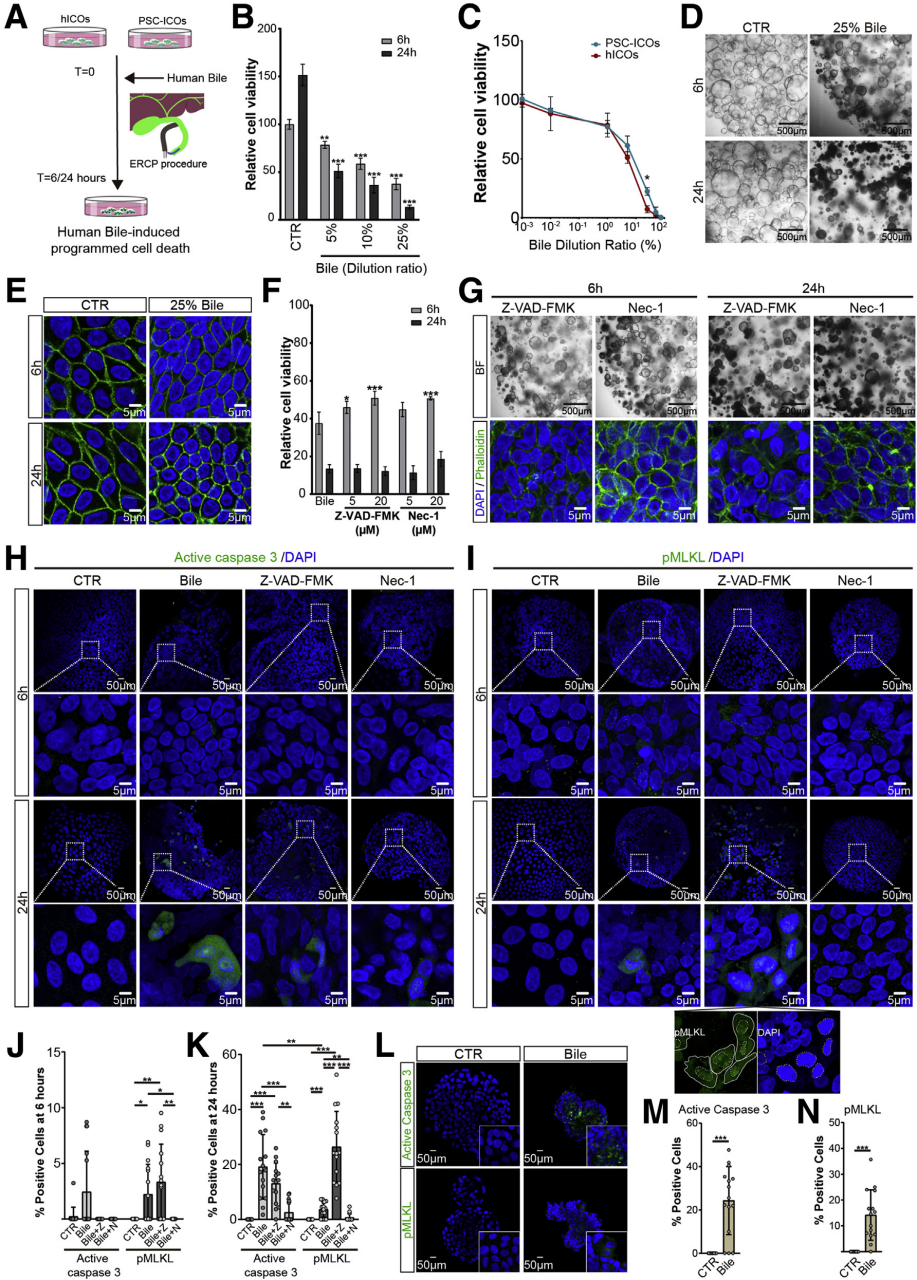
**Cell death induced by human bile in hICOs.** (*A*) hICOs and PSC-ICOs were exposed to ERCP-obtained human bile diluted in expansion medium at different ratios for 6 and 24 hours. (*B*) Cell viability of treated hICOs was measured using CellTiter-Glo reagent. Relative cell viability was normalized to untreated hICOs (CTR). (*C*) hICOs and PSC-ICOs were exposed to human bile for 24 hours at different dilution ratios. Cell viability was measured and analyzed as indicated. (*D*) Representative bright-field images of treated hICOs treated with human bile. (*E*) Confocal imaging of the treated hICOs after staining with 4′,6-diamidino-2-phenylindole (DAPI)/phalloidin. (*F*) hICOs were pretreated with Z-VAD-FMK or Nec-1 at the indicated concentration 1 hour before human bile treatment and then co-incubated with human bile (25%). Cell viability was determined and presented as mentioned earlier. (*G–K*) hICOs were pretreated with Z-VAD-FMK (20 μmol/L) or Nec-1 (20 μmol/L), respectively, and then incubated with human bile (25%) as indicated earlier. (*G*) Representative bright-field and confocal images (DAPI/phalloidin) of the treated hICOs are shown. (*H* and *I*) Staining of active caspase 3 and pMLKL in stimulated hICOs is shown. Enlarged images from the *boxed area* are shown in the *bottom panel*. Images with individual colors are shown below the indicated merged images (magnification, 1200×). (*I*). DAPI and pMLKL staining are marked by yellow and white outlines separately. Quantification of positive cells at (*J*) 6 and (*K*) 24 hours in fluorescent images was performed as indicated earlier. (*L–N*) PSC-ICOs were treated with human bile (25%) for 24 hours. (*L*) Staining of active caspase 3 and pMLKL in stimulated PSC-ICOs is shown. (*M* and *N*) Quantification of positive cells in fluorescent images was performed as indicated earlier. Data are means ± SD. ∗*P* < .05, ∗∗*P* < .01, ∗∗∗*P* < .001, (*B* and *F*) Mann–Whitney test, (*C*) 2-way analysis of variance post-Sidak multiple comparisons test, (*J* and *K*) Kruskal–Wallis test followed by the Dunn post hoc test, and (*M* and *N*) unpaired Student *t* test.

Cholangiocytes form the biliary epithelium and play a major role in fluidizing and alkalinizing canalicular bile. In physiological conditions, the apical cholangiocyte surface is protected against the hydrophobic and toxic bile by the biliary HCO_3_^-^ umbrella.^30^ The cytotoxicity of bile on cholangiocytes could be caused by cholestasis, change of bile composition, or impairment of the apical HCO_3_^-^ umbrella in pathologic status, which is associated with sclerosing cholangitis and biliary fibrosis.^31^ Direct cytotoxicity of bile acid could lead to cholangiocyte death, representing critical pathomechanism of cholangiopathies.^32^ Here, we investigated the direct cytotoxicity of human bile, collected during the endoscopic retrograde cholangiopancreatography (ERCP) procedure, in the hICOs and PSC-ICOs (Figure 7*A*). As shown in Figure 7*B*, bile exposure caused a significant time- and concentration-dependent reduction of cell viability in hICOs (n = 3; *P* < .01). In general, the hICOs and PSC-ICOs showed similar sensitivity to bile toxicity except for the concentration of 25% bile, in which PSC-ICOs appear to be more resistant to bile toxicity (n = 3; *P* < .05). The hICOs showed a time-dependent morphologic change featuring shrunken dark but intact lumen, diminished cytoplasm, complete actin cytoskeleton, and relatively reduced nucleus with irregular shapes, after bile exposure (Figure 7*D* and *E*), which are in agreement with the response of hICOs treated with gallbladder bile by Sampaziotis et al.^33^ Co-incubation with Z-VAD-FMK modestly reduced the bile-induced cell death at 6 hours (n = 3; *P* < .05), but did not affect 24-hour incubation (Figure 7*F* and *G*). Supplementing with Nec-1 showed a similar modest protective effect (Figure 7*F* and *G*). As shown in Figure 7*H*, *J*, and *K*, incubation with bile activated caspase 3 in hICOs at 24 hours, suggesting an induction of apoptosis (*P* < .001). The apoptosis could be reduced significantly by Nec-1 (*P* < .001), but not Z-VAD-FMK (*P* = .42) (Figure 7*I* and *K*). Sporadic pMLKL-positive cells were observed in bile-treated hICOs at 6 and 24 hours of incubation (*P* < .001), which was less than observed with active caspase 3–positive cells (3.705 ± 2.795 vs 19.14 ± 11.75 %; *P* < 0.01), and could be inhibited substantially by Nec-1 (*P* < .001) (Figure 7*I*, *J*, and *K*). Of note, supplementing with caspase inhibitor resulted in a significant increase of cytoplasmic and nuclear pMLKL after 24 hours of bile treatment (*P* < .001), implying a shift to necroptosis (Figure 7*I* and *K*). Activation of caspase 3 and phosphorylation of MLKL also could be found in bile-treated PSC-ICOs (*P* < .001) (Figure 7*L*, *M* and *N*). Together, these data show that human bile–induced cholangiotoxicity could be recapitulated in human ICOs, in which necroptosis is partially involved and can be exacerbated by caspase inhibition.

### Concentration-, Species- and Donor-Specific Responses of Necroptosis Inhibitors

**Figure 8:**
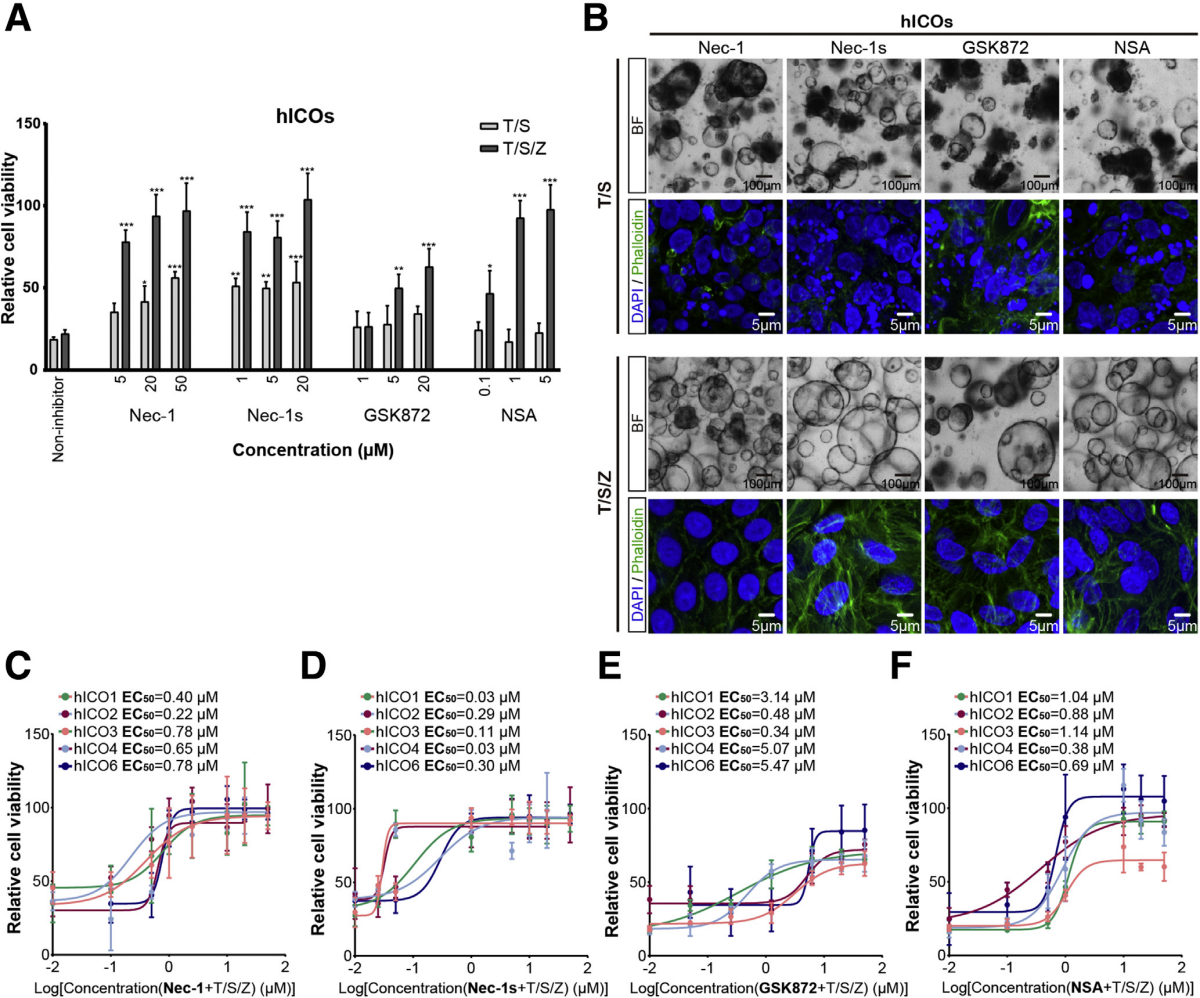
**Concentration- and donor-specific responses of necroptosis inhibitors.** (*A*) hICOs (n = 3) were exposed to T/S or T/S/Z for 12 hours, in the presence or absence of inhibitors at different concentrations. Cell viability was measured using CellTiter-Glo reagent. Relative cell viability was normalized to untreated hICOs (CTR). (*B*) T/S- and T/S/Z-exposed hICOs were co-incubated with Nec-1 (20 μmol/L), Nec-1s (20 μmol/L), GSK872 (20 μmol/L), or NSA (50 μmol/L) for 12 hours. Representative bright-field images and fluorescent images with 4′,6-diamidino-2-phenylindole (DAPI)/phalloidin staining are shown. (*C*, *D*, *E*, and *F*) hICOs (n = 5) were co-incubated with T/S/Z and the indicated necroptosis inhibitors for 12 hours. Drug response curves are shown. Data are means ± SD. (*A*) ∗*P* < .05, ∗∗*P* < .01, ∗∗∗*P* < .001, Kruskal–Wallis test followed by the Dunn post hoc test. BF, bright field.

The inhibitory effect of the well-defined necroptosis inhibitors, Nec-1, 7-Cl-O-Nec-1 (Nec-1s), GSK872, and necrosulfonamide (NSA), was examined in hICOs treated with T/S or T/S/Z (n = 3). Necroptosis in hICOs could be completely prevented by Nec-1 (50 μmol/L; *P* < .001) and Nec-1s (20 μmol/L; *P* < .001), and partly prevented by GSK872 (20 μmol/L; *P* < .001) and NSA (5 μmol/L; *P* < .001) (Figure 8*A*). These results were confirmed further by morphologic evaluation (Figure 8*B*). These last 2 inhibitors had no significant effect on the prevention of apoptosis in the T/S condition (Figure 8*A*). Further experiments were performed with hICOs derived from 5 donors and a detailed range of inhibitor concentrations. As shown in Figure 8*C–F*, donor-specific responses to necroptosis inhibitors were observed. The least variation was observed in Nec-1 (Figure 8*C*). For Nec-1s, hICO1 and 4 showed an obviously lower half-maximal effective concentration compared with the other 4 hICOs (Figure 8*D*). Similar donor-specific variances also were observed in GSK872 and NSA (Figure 8*E* and *F*).

**Figure 9:**
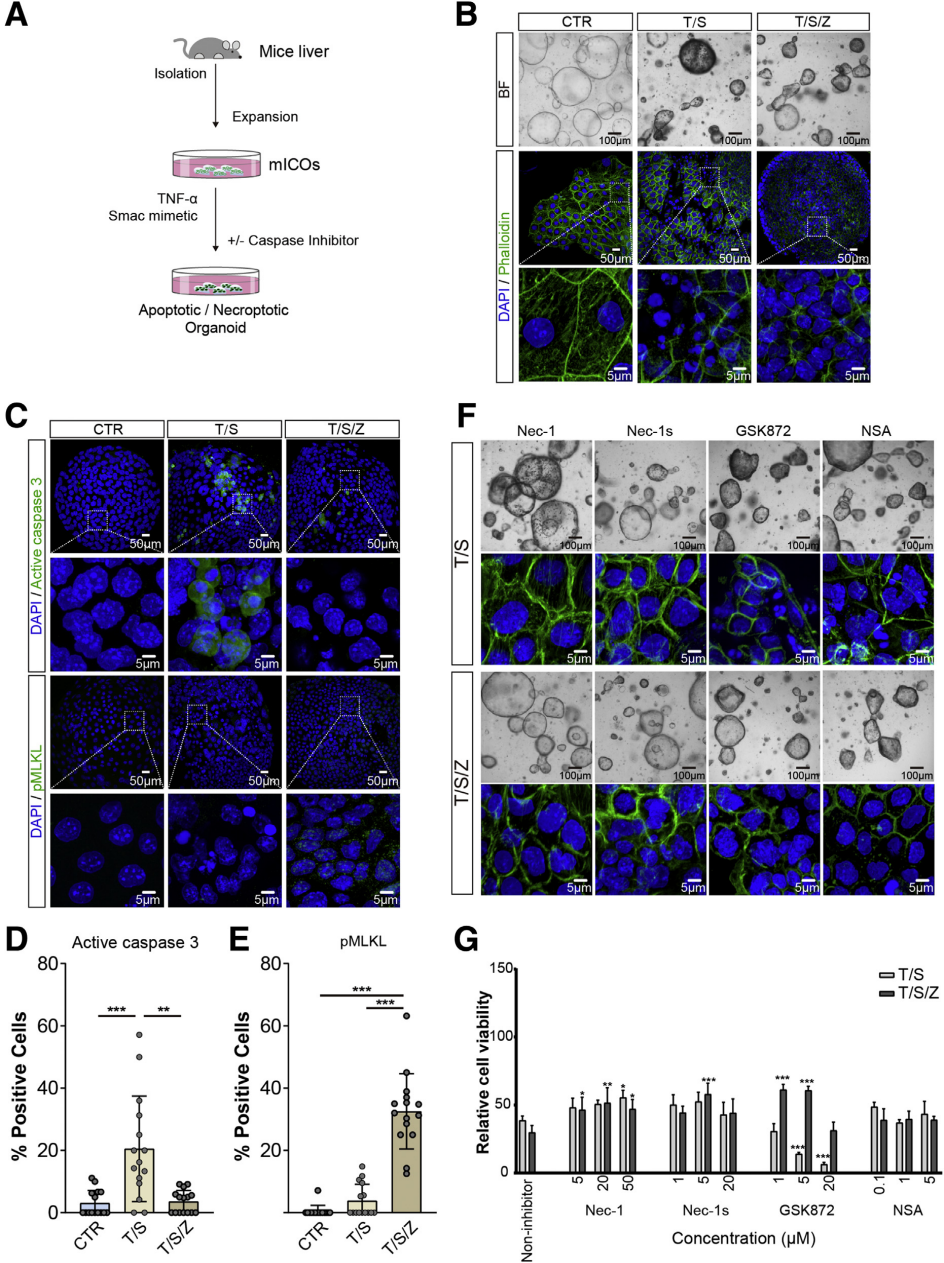
**Murine-specific apoptosis and necroptosis responses and drug sensitivity in mICOs.** (*A–C*) mICOs were isolated and cultured from mouse livers. mICOs were treated with TNF-α (T, 20 ng/mL) and Smac mimetic (S, 120 μmol/L), with or without Z-VAD-FMK (Z, 50 μmol/L), for 12 hours. (*B*) Representative bright-field images and fluorescent images with 4′,6-diamidino-2-phenylindole (DAPI)/phalloidin staining are shown. Enlarged images from the *boxed area* are shown in the *bottom panels*. (*C*) Staining of active caspase 3 and pMLKL in stimulated mICOs are shown. Enlarged images from the *boxed area* are shown in the *bottom panel*. (*D* and *E*) Quantification of active caspase 3– and pMLKL-positive cells in fluorescent images was performed using ImageJ (at least 15 high-power fields in each condition). (*F*) T/S- and T/S/Z-exposed mICOs were co-incubated with Nec-1 (20 μmol/L), Nec-1s (20 μmol/L), GSK872 (20 μmol/L), or NSA (50 μmol/L) for 12 hours. Representative bright-field images and fluorescent images with DAPI/phalloidin staining are shown. (*G*) mICOs (n = 3) were exposed to T/S or T/S/Z for 12 hours, in the presence or absence of inhibitors at different concentrations. Cell viability was measured using CellTiter-Glo reagent. Relative cell viability was normalized to untreated mICOs (CTR). hICOs (n = 4) were co-incubated with T/S/Z and indicated necroptosis inhibitors for 12 hours. Drug response curves are shown. Data are means ± SD. ∗*P* < .05, ∗∗*P* < .01, ∗∗∗*P* < .001, by (*G*, *D* and *E*) the Kruskal–Wallis test followed by the Dunn post hoc test. BF, bright field.

Necroptosis inhibitors show divergent potency and specificity among species.^6^^,^^13^ We further tested whether murine intrahepatic cholangiocyte organoids (mICOs) show differences in drug response compared with human hICOs. To this end, mICOs were exposed to apoptotic and necroptotic stimuli described in hICOs (Figure 9*A*). The cell death induced by T/S and T/S/Z shared similar morphologic features in human hICOs (Figure 9*B*). Immunostaining of active caspase 3 and pMLKL further verified the apoptosis and necroptosis induced in mICOs (Figure 9*C–E*). Regarding the necroptosis inhibitors, we found that NSA failed to prevent apoptosis or necroptosis in mICOs (Figure 9*F* and *G*). Both Nec-1 and Nec-1s could attenuate necroptosis significantly in mICOs (n = 3; *P* < .05), but at a lower efficiency than in hICOs (Figure 9*G*). Apoptosis induced in mICOs also could be rescued by Nec-1 (n = 3; *P* < .05), but not Nec-1s (Figure 9*G*). Surprisingly, GSK872 showed a protective effect against necroptosis at low concentrations, but exacerbated apoptosis instead (n = 3; *P* < .001), suggesting a potential divergent programmed cell death machinery in mice from that in human beings (Figure 9*F* and *G*). Together, these data show species- and donor-specific variations of human and murine cholangiocyte organoids in response to necroptosis inhibitors.

### Drug Screening Using hICOs Identifies Dabrafenib as a Potent Necroptosis Inhibitor

**Figure 10:**
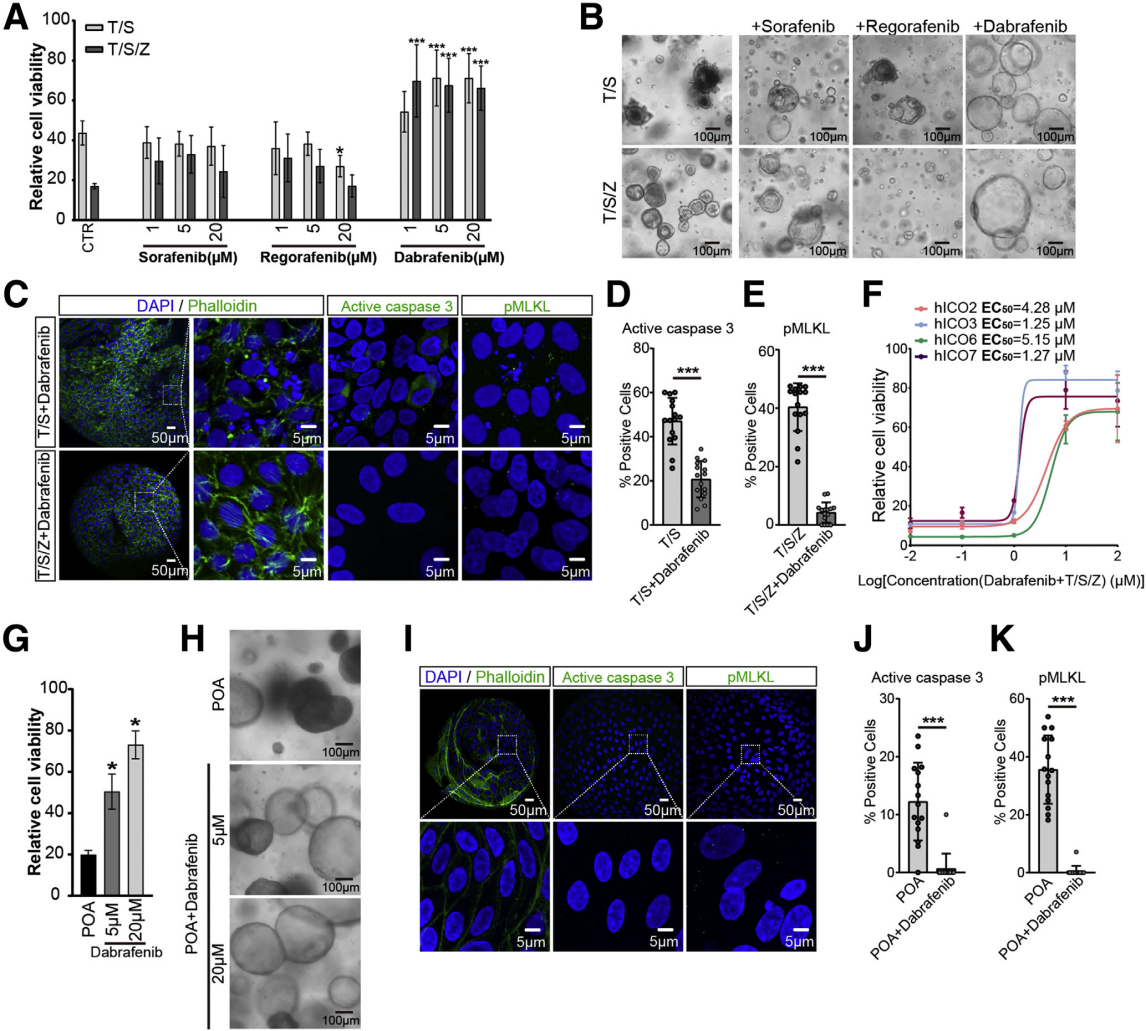
**Drug screening using hICOs identifies dabrafenib as a potent necroptosis inhibitor.** (*A–C*) hICOs were exposed to T/S or T/S/Z for 6 hours, in the presence or absence of sorafenib, regorafenib, or dabrafenib at different concentrations (n = 6). (*A*) Cell viability was measured using CellTiter-Glo reagent. Relative cell viability was normalized to untreated hICOs (CTR). (*B*) hICOs were treated with T/S or T/S/Z for 6 hours, supplemented with sorafenib (20 μmol/L), regorafenib (20 μmol/L), or dabrafenib (20 μmol/L). Representative bright-field images are shown. (*C*) T/S/Z-treated hICOs were co-incubated with dabrafenib (20 μmol/L) for 6 hours. Representative fluorescent images with 4′,6-diamidino-2-phenylindole (DAPI)/phalloidin, active caspase 3, and pMLKL staining are shown. (*D* and *E*) Quantification of positive cells in fluorescent images was performed using ImageJ (at least 15 high-power fields in each condition). (*F*) hICOs (n = 5) were co-incubated with T/S/Z and indicated dabrafenib for 12 hours. Drug response curves are shown. (*G–K*) hICOs were pretreated with dabrafenib (5/20 μmol/L) 1 hour before POA treatment and then co-incubated with POA (1.6 mmol/L) for 6 hours (n = 4). (*G*) Cell viability was measured and analyzed as mentioned earlier. (*H*) Representative bright-field images are shown. (*I*) Representative fluorescent images with DAPI/phalloidin, active caspase 3, and pMLKL staining are shown. Enlarged images from the *boxed area* are shown in the *bottom panel*. (*J* and *K*) Quantification of positive cells in fluorescent images was performed using ImageJ (at least 15 high-power fields in each condition). Data are means ± SD. ∗*P* < .05, ∗∗∗*P* < .001, by (*G*) Mann–Whitney test, (*D*, *E*, *J*, and *K*) unpaired Student *t* test, and (*A*) the Kruskal–Wallis test followed by the Dunn post hoc test.

Next, we deployed necroptotic hICOs as a preclinical drug screening tool to identify necroptosis inhibitors. We tested several Food and Drug Administration–approved kinase inhibitors in clinical practice as targeted cancer therapies, including sorafenib, regorafenib, and dabrafenib. These drugs were suggested to have antinecroptosis potential.^34^ We found that only dabrafenib showed dose-depended inhibition of necroptosis in hICOs (n = 6; *P* < .001) (Figure 10*A* and *B*). Dabrafenib also showed clear anti-apoptosis potential (n = 6; *P* < .001) (Figure 10*A* and *B*). Treatment with dabrafenib resulted in suppression of both caspase 3 activation and MLKL phosphorylation (Figure 10*C–E*). Interestingly, similar to Nec-1s, GSK872, and NSA, a donor-specific drug response also was found in dabrafenib, in which hICO3 and hICO7 showed obviously lower half-maximal effective concentration values compared with hICO2 and hICO6 (Figure 10*F*). To further evaluate the therapeutic potential of dabrafenib in preclinical models, it was tested in ethanol-metabolite– and bile-induced cholangiocellular toxicity. Dabrafenib significantly attenuated POA-induced cell death (n = 4; *P* < .05) (Figure 10*G* and *H*) by completely inhibiting both the caspase 3 activation and MLKL phosphorylation (Figure 10*I–K*). Dabrafenib failed to prevent bile-induced cell death (data not shown). Overall, these data point to a preclinical application of hICOs as a drug screening tool for cell death inhibitors.

## Discussion

The critical role of necroptosis in liver diseases such as NASH, ALD, and drug- or ischemia-induced liver injury has been well established.^35^ Nonetheless, current studies regarding the pathogenic role of necroptosis in cholangiopathies are scarce. Compelling information shows that activation of necroptosis upon hepatic insults largely relies on the availability of RIPK3.^9^^,^^10^ RIPK3 was expressed predominantly in cholangiocytes rather than hepatocytes in NASH and PBC patients.^9^^,^^11^ Consistent with these findings, we hereby show that RIPK3 is expressed primarily in cholangiocytes in patients with NASH, PSC, and ALD, and recipients undergoing liver retransplantation. Notably, RIPK3 also has been found in cholangiocytes in the ductal reaction area and part of hepatocytes adjacent to fibrotic areas, possibly resulting from the suppressive role of RIPK3 in hepatic compensatory proliferation upon chronic hepatic insults.^36^ Likewise, pMLKL shared a similar biliary expression pattern in some, but not all, patients with end-stage liver disease, suggesting that necroptosis mainly contributes to biliary injury in chronic liver diseases. Regarding liver transplantation, pMLKL, but not RIPK3, has been observed predominately in periportal nonparenchymal cells, other than cholangiocytes or hepatocytes, in donor livers, probably implying the involvement of necroptosis in hepatic ischemia-reperfusion injury. These findings provide direct clinical evidence of necroptosis involvement in cholangiopathies.

With the establishment of a culture method for human liver-derived cholangiocyte organoids, it has become possible to model liver and particular bile duct diseases for drug discovery. To date, the preclinical application of either donor- or patient-derived ICOs in cholangiopathy study has been limited. Cholangiopathies are inflammatory diseases in which proinflammatory chemokines and cytokines, such as TNF-α, represent leading pathogenic factors.^8^ In this study, we successfully recapitulated canonical TNF-α–mediated necroptosis in hICOs, as well as ALD-ICOs and PSC-ICOs. Intriguingly, despite evident activation of caspase 8 and caspase 3, apoptotic ICOs also were featured by pMLKL expression confined in the nucleus, but not the cytoplasm. Given that cytoplasmic and membrane translocation of pMLKL is widely regarded as the major functional form of MLKL for necroptosis execution, the nucleus-located pMLKL in apoptotic ICOs might suggest an event possibly serving a unique role that is not associated with necroptosis.^37^ In addition, Cao et al^26^ showed that nuclear translocation of MLKL functions as an effector of endoplasmic reticulum stress-related apoptosis, which might be a potential elucidation.

Recent studies uncovered that necroptotic cells orchestrate innate and adaptive immunity by passive DAMPs released from damaged cells and activation of NF-κB–dependent transcriptional responses that occur simultaneously with necroptosis.^7^ The necroptosis modeled in hICOs by TNF-α stimulation represent experimental and inflammation-associated cholangiocellular injury in vitro. Our data highlight the up-regulation of a panel of necroptosis-associated cytokines/chemokines in necroptotic hICOs compared with apoptotic or TNF-α–treated hICOs, which could be down-regulated significantly by supplementing with Nec-1. Furthermore, concomitant activation of NF-κB signaling occurred in both apoptotic and necroptotic hICOs, but in distinct manners. These findings confer hICOs as a promising tool to study cholangiopathies, particularly with an eye on biliary inflammation. However, limitations remain in our study owing to the lack of inflammatory cells in the organoid system. Future studies performed on a co-culture system comprising hICOs and immune cells could facilitate research of the immunogenetic properties of programmed cell death in cholangiopathies.

The TNF-α–mediated necroptosis and apoptosis modeled in ICOs provide a set of methodologies for evaluating and validating cell death signaling in 3-dimensional culture. In light of that, we further sought to establish proof-of-concept cholangiopathy models in ICOs for toxicity studies, with particular insults leading to cholangiopathies. Nonoxidative metabolites of ethanol, such as POA, are the major causative agents of ductal cell damage resulting from alcohol abuse by reducing the stability of cystic fibrosis transmembrane conductance regulator, triggering calcium overload, and ultimately giving rise to epithelial necrosis.^29^^,^^38^ Interestingly, we found that POA could induce extensive necroptosis in hICOs and ALD-ICOs, which is in line with a recent study on pancreatic acinar cells.^39^ Nec-1 showed a completely protective property preventing POA-induced injury, implying a potential application of necroptosis inhibitor in ethanol-associated biliary injury. Of note, different from the biliary epithelium in the human body, the cell polarity in ICO culture is organized reversely, in which the basolateral side is at the extraluminal surface, directly contacting the toxic insults.^40^ It has been reported that the basolateral side of the cholangiocyte is more susceptible to toxin stimulations owing to the lack of barrier.^30^^,^^41^ Therefore, to better recapitulate cholangiocyte responses to various toxins, an optimized tissue-engineered bile duct, resembling critical biliary physiological functions, should be explored in the future.

Although not clarified yet, it commonly is suggested that toxic bile causes cholangiocellular damage in cholestatic liver diseases by mediating cell death.^32^ Currently, compared with hepatocytes, much less is known about the bile toxicity on human biliary epithelial cells. Increased apoptotic bile duct loss in PBC and PSC patients has been reported in several studies.^42^, ^43^, ^44^ Limited studies have uncovered the molecular mechanism of bile-induced cholangiocyte apoptosis in vitro.^30^^,^^45^ Here, we showed that human bile could evoke apparent cell death in hICOs and PSC-ICOs, which are possibly the mixture of apoptosis and necroptosis, and only partially could be restored by either caspase inhibitor or Nec-1. Inversely, instead of rescuing the human bile–induced apoptosis, the supplement of Z-VAD-FMK evoked substantial necroptosis in hICOs. Of note, caspase inhibition is a common mechanism used by viruses to prevent host cell apoptosis in viral liver diseases. This raises the possibility that necroptosis also could be involved in bile-mediated biliary damage, particularly in the presence of viral infection. We and others previously described that cholangiocyte damage caused by rotavirus infection might be involved in the pathogenesis of biliary atresia, in which necroptotic signaling was activated in rotavirus-infected hICOs.^46^^,^^47^ The strong immunogenic nature of necroptosis may provide new insight into the extensive inflammation in biliary atresia or cholangitis in viral diseases. The interaction among bile toxicity, virus infection, and cholangiocyte damage is worth further investigation.

In this study, although hICOs, as well as ALD-ICOs and PSC-ICOs, showed interdonor variations in the sensitivity to cell death induction in the early stage, they ultimately committed to identical cell death, suggesting that liver disease etiologies might not be able to significantly influence the cell death program in ICOs. Considering the availability and intricate disease background of patient-derived organoids, hICOs appear to be a better option for the study of cholangiopathy-associated programmed cell death in vitro. Moreover, organoids derived from individual donors hold great promise for use in drug screening and discovery. Intriguingly, the potency of well-defined necroptosis inhibitors and dabrafenib varied largely in different donors, suggesting that hICOs could be applied as a drug screening tool for personalized therapy. In addition, the effectiveness of NSA is reported to be species-specific as a result of the divergent MLKL pseudokinase domains between species.^6^^,^^48^ Our data show that NSA could protect only hICOs, but not mICOs. GSK872, a RIPK3 inhibitor, showed reverse effects in necroptotic hICOs and mICOs, suggesting a species-specific response of necroptosis inhibitors.

The clinical use of experimental necroptosis inhibitors has been greatly hampered by safety issues and a short half-life. Recent studies have shown that several Food and Drug Administration–approved kinase inhibitors, widely used in clinical practice as anticancer drugs, exert an inhibitory capacity against necroptosis.^34^ In this study, sorafenib, dabrafenib, and regorafenib were tested, and only dabrafenib showed prevention of necroptosis, diverging from previous results.^34^ Interestingly, we also found that dabrafenib can rescue apoptotic hICOs effectively, possibly owing to its specific RIPK3-targeting potential. Moreover, dabrafenib effectively could rescue hICOs exposed to POA by inhibiting both apoptosis and necroptosis, suggesting a broad-spectrum cell death inhibitory capacity in ethanol-associated cholangiocellular damage.

In conclusion, our study shows that the human cholangiocyte organoids can recapitulate cholangiopathy-associated necroptosis signaling in vitro. This provides a new preclinical model that can be used for drug screening. These findings may have implications for the future development of therapeutics for the treatment of cholangiopathies.

## Methods

### Patients, Specimens, and Materials

For immunohistochemistry staining, explant liver biopsy specimens, collected during liver transplantation procedures at the Erasmus Medical Center, were obtained from patients with chronic liver diseases including PSC (n = 3), ALD (n = 3), and NASH (n = 3). Biopsy specimens from recipients undergoing liver retransplantation (n = 3) owing to hepatic artery thrombosis, ischemic-type biliary lesions, and primary graft nonfunction also were enrolled. Furthermore, liver specimens from donation after cardiac death donors (n = 3) and donation after brain death donors (n = 3) were collected during transplantation at 60 minutes after portal reperfusion. Donor liver sample from LDLT (n = 1) was collected as the ischemia-free control for immunohistochemistry. Patient and donor demographic and clinical characteristics are summarized in Table 1. For initiation of organoid cultures, fresh liver biopsy specimens, including donor livers (n = 7) and explant livers from ALD (n = 3) and PSC (n = 3) patients, were collected at the time of transplantation and stored in cold University of Wisconsin solution. The demographic characteristics of donors and patients are listed in Table 2. For organoid exposure to bile toxicity, fresh bile samples were collected during therapeutic ERCP (n = 3). To limit interpatient variations in bile composition, bile samples were pooled by mixing equal parts. Patient demographic and clinical characteristics are summarized in Table 3. The use of these human samples was approved by the Erasmus MC medical ethics council (MEC-2014-060, MEC-2016-743, and MEC 2018-1174), and all patients gave informed consent for the use of material for research purposes. The Committee on the Ethics of Animal Experiments of the Erasmus Medical Center approved the use of animal specimens.

**Table 1:** The Main Demographic and Clinical Characteristics of Patients and Donors for Immunohistochemistry Analysis

	LDLT donor	DBD donor			DCD donor			Reltx			PSC			ALD			NASH		
	D1	D1	D2	D3	D1	D2	D3	P1	P2	P3	P1	P2	P3	P1	P2	P3	P1	P2	P3
Sex, F/M	F	F	M	M	F	M	F	M	M	F	M	M	M	M	M	F	M	F	M
Age, *y*	26	55	65	60	58	49	58	34	42	38	21	49	47	63	65	43	68	52	53
BMI, *kg/m*^*2*^	23.62	24.22	23.15	24.93	25.01	24.38	23.88	26.45	23.1	39.84	23	18.56	30.18	16.9	34.57	21.13	26.04	32.82	26.32
AST level, *U/L*	20	19	30	26	29	124	57	253	73	2557	54	93	141	39	50	59	40	144	146
ALT level, *U/L*	36	18	58	59	24	158	47	1520	51	914	28	61	76	63	31	34	33	73	88
GGT level, *U/L*	11	13	202	120	61	70	54	258	583	84	90	116	104	150	329	49	183	21	179
ALP level, *U/L*	37	65	95	57	57	53	58	75	1122	98	379	194	506	179	124	128	86	213	375
Total bilirubin level, *mg/dL*	8	3.6	15	10.1	4	6	10	62	146	296	66	65	213	32	20	57	12	61	293
INR	1	1.25	1.1	–	–	1.1	1	1.5	3	2	1.3	1.5	1.4	1.7	1.1	2	1.1	1.7	1.7
HBV, yes/no	N	N	N	N	N	N	N	N	N	N	N	N	N	N	N	N	N	N	N
HCV, yes/no	N	N	N	N	N	N	N	N	N	N	N	N	N	N	N	N	N	N	N
MELD score	–	–	–	–	–	–	–	20	27	38	14	18	20	15	8	19	7	17	27

ALP, alkaline phosphatase; ALT, alanine aminotransferase; AST, aspartate aminotransferase; BMI, body mass index; D, donor; DBD, donation after brain death; DCD, donation after cardiac death; F, female; GGT, γ-glutamyltransferase; HBV, hepatitis B virus; HCV, hepatitis C virus; INR, international normalized ratio; M, male; MELD, model for end-stage liver disease; P, patient; Reltx, liver retransplantation.

**Table 2:** The Main Demographic Features of Donors and Patients with Biopies Collected for ICOs Initiation

	hICOs (n = 7)	PSC-ICOs (n = 3)	ALD-ICOs (n = 3)	*P* value
Age, *y*, average ± SD	42.1 ± 14.7	46.0 ± 8.2	58.6 ± 4.9	NS
Sex, % male	14%	100%	100%	.029
BMI, *kg/m*^*2*^, average ± SD	30.9 ± 6.7	20.7 ± 0.3	24.0 ± 2.2	NS

BMI, body mass index.

**Table 3:** The Main Demographic Features of Patients Undergoing ERCP With Bile Collected

No.	Age, *y*	Sex	BMI, *kg/m*^*2*^	Diagnosis
ERCP1	79	Female	20.9	Extrahepatic cholangiocarcinoma
ERCP2	20	Male	23.88	Primary sclerosing cholangitis
ERCP3	67	Male	23.36	Anastomotic biliary strictures after liver transplantation

BMI, body mass index.

### Human and Murine Cholangiocyte Organoid Initiation and Culture

The hICOs (n = 7), ALD-ICOs (n = 3), and PSC-ICOs (n = 3) were initiated and cultured as previously described.^16^^,^^17^ Briefly, liver tissue biopsy specimens (0.5–1 cm^3^) derived from donors’ or patients’ livers were minced, rinsed twice with Earle's balanced salt solution (Thermo-Fisher, Waltham, MA), and digested in collagenase solution (2.5 mg/mL collagenase Type A; Sigma-Aldrich, St. Louis, MO) in advanced Dulbecco’s modified Eagle medium (DMEM)/F-12 medium (Invitrogen, Carlsbad, CA) for 15 minutes at 37°C. The digestion was stopped by the addition of cold advanced DMEM/F-12, and then the single-cell suspension was filtered through a 70-μm nylon cell strainer and spun at 1500 rpm for 5 minutes at 4°C. The cell pellets were collected and mixed with cold Basement Membrane Extract, Type 2 (R&D Systems, Minneapolis, MN). Cells then were seeded in 25-μL droplets in multiwell plates. After Basement Membrane Extract was solidified, a 500-uL initiating medium was added, and cells were cultured at 37°C in a humidified atmosphere with 5% CO_2_. The initiating medium was based on advanced DMEM/F-12 medium, supplemented with multiple components listed in Table 4. After 3 days of culture, the medium was refreshed and switched to expansion medium, in which noggin, Wnt, Y27632, and human embryonic stem (hES) cell cloning recovery supplement were deprived. The medium was refreshed every 2–3 days, and organoids were passaged in a 1:3 split ratio every 7 days. Healthy mICOs were isolated from C57BL/6 mouse liver and cultured as described previously.^15^

**Table 4:** List of Media Compositions Used in Cholangiocyte Organoid Cultures in This Study

Components	Concentration	Source
N2	1%	Gibco (Amarillo, TX)
B27	1%	Gibco
N-acetylcysteine	1.25 mmol/L	Sigma-Aldrich
Gastrin	10 nmol/L	Sigma-Aldrich
Epidermal growth factor	50 ng/mL	Peprotech
Fibroblast growth factor 10	100 ng/mL	Peprotech
Hepatocyte growth factor	25 ng/mL	Peprotech
R-spondin	10%	Conditioned medium
Nicotinamide	10 nmol/L	Sigma-Aldrich
A83.01	5 μmol/L	Tocris
Forskolin	10 μmol/L	Tocris
Noggin	25 ng/mL	Conditioned medium
Wnt	30%	Conditioned medium
Y27632	10 μmol/L	Sigma-Aldrich
hES cell cloning recovery supplement	2 μmol/L	Stemgent

### Cell Death Induction

Cell death was induced and inhibited in both hICOs and mICOs by incubation with various chemicals and ERCP-obtained bile at different concentrations. To induce cell death, hICOs and mICOs were treated with recombinant human TNF-α (T) (20 ng/mL; Peprotech, London, UK), Smac mimetic (S) (120 μmol/L, LCL-161; Selleckchem, Houston, TX), and Z-VAD-FMK (Z) (50 μmol/L; InvivoGen, San Diego, CA). POA (Sigma-Aldrich) was dissolved in ethanol and used in hICOs and ALD-ICOs (0.2–1.6 mmol/L) to mimic ethanol-induced injury. ERCP-obtained bile was diluted in expansion medium and used in hICOs and PSC-ICOs to mimic bile-induced damage. To inhibit the induced cell death, Nec-1 (5–50 μmol/L; Sigma), Nec-1s (1–20 μmol/L; Selleckchem), GSK872 (1–20 μmol/L; Abcam, Cambridge, MA), NSA (0.1–5 μmol/L: Tocris, Abingdon, UK), dabrafenib (1–20 μmol/L; Medchemexpress, Monmouth Junction, NJ), sorafenib (1–20 μmol/L; Santa Cruz Biotechnology, Santa Cruz, CA), and regorafenib (1–20 μmol/L; Selleckchem) were assessed. Stimulated hICOs were stained with PI (Sigma-Aldrich) according to the manufacturer's instructions. Briefly, hICOs were refreshed with prewarmed (37°C) advanced DMEM/F-12 medium supplemented with 100 μg/mL PI. Organoids then were incubated in the dark at 37°C for 30 minutes, and bight-field and fluorescent images were captured using the EVOS cell imaging system (Thermo-Fisher). The average diameter of stimulated organoids was determined in bight-field images (at least 25 organoids per subgroup) in triplicate using ImageJ software (National Institutes of Health, Bethesda, MD) as previously described by Sampaziotis et al.^49^

### Cell Viability Assay

According to the manufacturer's instructions, the CellTiter-Glo 3D Cell Viability Assay (Promega, Madison, WI) was applied to determine the viability of stimulated organoids. Briefly, trypsinized organoids were seeded into wells of a 96-well plate at a concentration of 1.0 × 10^6^ cells/mL. After 3 days of culture, the organoids were treated with the cell death inducers and inhibitors, as indicated previously, and a volume of CellTiter-Glo reagent equal to the volume of cell culture medium was added to each well. The plates were mixed vigorously for 5 minutes and then incubated at room temperature for 25 minutes in the dark to stabilize the luminescent signal. The luminescent light units were recorded by a microplate reader (BMG Labtech, Durham, NC).

### Real-Time Quantitative Reverse-Transcription Polymerase Chain Reaction

hICOs were collected in QIAzol lysing reagent (Qiagen, Duesseldorf, Germany), and total RNA was isolated using the miRNeasy mini kit (Qiagen). The concentration of RNA was measured with an Agilent 2100 Bioanalyzer (Agilent Technologies, Santa Clara, CA). Isolated RNA then was reverse-transcribed to complementary DNA using PrimeScript RT Master Mix reagent (Takara, Tokyo, Japan) according to the manufacturer's instructions. Real-time quantitative reverse-transcription polymerase chain reaction was performed using a Real-Time Polymerase Chain Reaction Detection System with SYBR Green incorporation (both from Bio-Rad Laboratories, Hercules, CA). *HPRT* and *GADPH* were used as housekeeping genes. The expression of a panel of genes was assessed, including *TNF-α*, *CCL20*, *CXCL8*, *IL1β*, *CXCL2*, and *CCL2*. The sequence of primers is listed in Table 5. The relative expression of genes was calculated as 2^−(ΔCt sample−ΔCt control)^. Each sample was evaluated at least in duplicate.

**Table 5:** List of Primer Sequences Used for Quantitative Polymerase Chain Reaction in This Study

Gene	Forward primer	Reverse primer
*TNF-α*	CTCTTCTGCCTGCTGCACTTTG	ATGGGCTACAGGCTTGTCACTC
*CCL20*	TGCTGTACCAAGAGTTTGCTC	CGCACACAGACAACTTTTTCTTT
*CXCL8*	GACCACACTGCGCCAACAC	CTTCTCCACAACCCTCTGCAC
*IL1β*	CTCGCCAGTGAAATGATGGCT	GTCGGAGATTCGTAGCTGGAT
*CXCL2*	ACCGAAGTCATAGCCACACTC	TCTTAACCATGGGCGATGCG
*CCL2*	GAGAGGCTGAGACTAACCCAGA	ATCACAGCTTCTTTGGGACACT
*HPRT*	ACCAGTCAACAGGGGACATAA	CTTCGTGGGGTCCTTTTCACC
*GADPH*	TCCTGTTCGACAGTCAGCCGC	CCAGGCGCCCAATACGACCA

### RNA Sequence Analysis

Raw sequencing reads of primary human hepatocytes (n = 2)^22^ and primary cholangiocytes derived from the common bile duct (CBD; n = 4)^23^ were extracted from bulk RNA sequencing profiles as described. The raw reads were uploaded to the Galaxy web platform (usegalaxy.eu, Freiburg im Breisgau, Germany) and mapped to the human reference genome (GRCh38.p13) using the RNA STAR tool (Galaxy, version 2.7.2b). Count reads were obtained using the “--quantMode GeneCounts” option in STAR. The heatmap was generated using the pheatmap package in R (v3.6.3, www.r-project.org).

### Immunostaining and Imaging

#### Paraffin-embedded liver tissue

Liver tissue biopsy specimens were fixed in 4% paraformaldehyde for 24 hours, followed by dehydration, paraffin embedding, and sectioning according to standard procedures. Immunohistochemistry was performed as previously described.^50^ In short, paraffin-embedded tissue sections (3-μm thick) were dewaxed and rehydrated via gradient ethanol washes. Antigen retrieval then was performed by heating the sections at 100°C in 10 mmol/L citrate acid buffer (pH 6.0). Non-specific staining was prevented by incubating with 1% bovine serum albumin and 10% normal goat serum (both from Sigma-Aldrich) in phosphate-buffered saline. The sections then were incubated with primary antibody diluted in 1% bovine serum albumin (Sigma-Aldrich) /0.025% Triton X-100 (Sigma-Aldrich)/1% normal goat serum (Abcam)(antibody diluent), at 4°C overnight. Information for all the primary antibodies used is listed in Table 6. Sections were incubated with 0.3% hydrogen peroxide for 15 minutes and by a 1-hour incubation with secondary goat anti-rabbit immunoglobulins/horseradish peroxidase (1:200; Dako, Glostrup, Denmark) at room temperature. The reaction products were visualized using a 3,3’-diaminobenzidine substrate kit (Dako). Sections incubated with dilution buffer without primary antibody were used as a negative control (data not shown). Images were acquired on NanoZoomer (Hamamatsu, Iwata City, Japan).

**Table 6:** List of Antibodies Used in This Study

Antibody	Reactivity	Application	Species		Source	Dilution
RIPK3	Human	IHC/IF	Rabbit	Polyclonal	Novus (NBP1-77299, Saint Charles, MO)	1:200
pMLKL	Human/mouse	IHC/IF	Rabbit	Polyclonal	Invitrogen (PA5-105678)	1:200
Active caspase 3	Human/mouse	IF	Rabbit	Polyclonal	R&D Systems (AF835)	1:40
Cleaved caspase 8	Human/mouse	IF	Rabbit	Monoclonal	Cell Signaling (9496S, Danvers, MA)	1:250
KRT19	Human	IHC	Mouse	Monoclonal	Dako (M088801-2)	1:50
p-IKKα/β	Human	IF	Rabbit	Monoclonal	Cell Signaling (2697S)	1:100
p–NF-κB2 p100	Human	IF	Rabbit	Polyclonal	Cell Signaling (4810S)	1:50
p65–NF-κB_active_	Human	IF	Mouse	Monoclonal	Sigma (MAB3026)	1:100

IF, immunofluorescence; IHC, immunohistochemistry; ; KRT19, cytokeratin-19.

#### Organoids

For histologic analysis, both hICOs and mICOs were washed with phosphate-buffered saline and fixed in 4% paraformaldehyde (15 minutes at room temperature), and stored in 1% paraformaldehyde–phosphate-buffered saline at 4°C for further whole-mount immunostaining. Briefly, non-specific antibody binding was blocked, and the organoids were incubated as described previously. Secondary goat anti-rabbit 488 or anti-mice 555 antibodies (both from Invitrogen) subsequently were diluted in a 1:1000 ratio and incubated with organoids for 1 hour at room temperature. Organoids were mounted using an antifade mounting medium with 4′,6-diamidino-2-phenylindole (Vector Labs, Burlingame, CA). F-actin was stained using Alexa Fluor phalloidin (Invitrogen) according to the manufacturer's instructions.

### Image acquisition and analysis

Fluorescent images were captured on a confocal microscope (LSM 700; Carl Zeiss, Jena, Germany) and further processed using ZEN software (Carl Zeiss). The laser intensity was kept the same for all samples. Fluorescent images were projected with maximum intensity and at least 15 areas with cells in each sample were selected. The positivity of fluorescent targets were measured using FIJI (Image J 1.51c, National Institutes of Health, Maryland, USA).

### TEM

To perform TEM, hICOs were fixed in 1.6% glutaraldehyde after treatment and stored at 4°C until processed. Subsequently, secondary fixation was performed using 1% osmium tetroxide for 1 hour. Next, acetone rinses were applied (8 times with an increased percentage of acetone for 15 minutes), and samples were infiltrated with 100% acetone/epoxy embedding medium (1:1) for 2 days. Samples were embedded for 1 hour at room temperature, followed by an embedding at 40°C overnight, and, finally, a 24-hour embedding at 60°C. Subsequently, ultrathin sections (60–70 nm) were created at a 6º angle at a speed of 1 mm/s with a Diatome Diamond knife (DiATOME, Hatfield, PA ) and Ultramicrotome Leica EM UC7 (Leica, Wetzlar, Germany). Finally, ultrathin sections were imaged using a Morgagni 268D microscope (FEI, Hillsboro, OR).

### Statistical Analysis

All statistical analyses were performed using Prism software (GraphPad Software, Inc, San Diego, CA). Data are expressed as the means ± SD from at least 3 replicates. The distribution of all data sets was analyzed for normality using the Shapiro–Wilk test. A comparison between the 2 groups was conducted using the unpaired Student *t* test or the Mann–Whitney test. Comparisons between multiple groups were performed using a 1-way analysis of variance test followed by the Tukey post hoc test or the Kruskal–Wallis test followed by the Dunn post hoc test or 2-way analysis of variance test followed by the post-Sidak test. Categoric variables were analyzed using the Fisher exact test. For each test, *P* < .05 was considered statistically significant.

All authors had access to the study data and reviewed and approved the final manuscript.
